# The Mitochondrial Enzyme 17βHSD10 Modulates Ischemic and Amyloid-β-Induced Stress in Primary Mouse Astrocytes

**DOI:** 10.1523/ENEURO.0040-22.2022

**Published:** 2022-10-05

**Authors:** Vanya Metodieva, Terry Smith, Frank Gunn-Moore

**Affiliations:** 1 Medical and Biological Sciences Building, School of Biology, North Haugh, University of St Andrews, St Andrews, Fife KY16 9TF, Scotland, United Kingdom; 2 Biomolecular Sciences Building, School of Biology, North Haugh, University of St Andrews, St Andrews, Fife KY16 9TF, Scotland, United Kingdom

**Keywords:** 17β-hydroxysteroid dehydrogenase type 10, Alzheimer’s disease, astrocytes, ischemia, metabolism, mitochondria

## Abstract

Severe brain metabolic dysfunction and amyloid-β accumulation are key hallmarks of Alzheimer’s disease (AD). While astrocytes contribute to both pathologic mechanisms, the role of their mitochondria, which is essential for signaling and maintenance of these processes, has been largely understudied. The current work provides the first direct evidence that the mitochondrial metabolic switch 17β-hydroxysteroid dehydrogenase type 10 (17βHSD10) is expressed and active in murine astrocytes from different brain regions. While it is known that this protein is overexpressed in the brains of AD patients, we found that 17βHSD10 is also upregulated in astrocytes exposed to amyloidogenic and ischemic stress. Importantly, such catalytic overexpression of 17βHSD10 inhibits mitochondrial respiration during increased energy demand. This observation contrasts with what has been found in neuronal and cancer model systems, which suggests astrocyte-specific mechanisms mediated by the protein. Furthermore, the catalytic upregulation of the enzyme exacerbates astrocytic damage, reactive oxygen species (ROS) generation and mitochondrial network alterations during amyloidogenic stress. On the other hand, 17βHSD10 inhibition through AG18051 counters most of these effects. In conclusion, our data represents novel insights into the role of astrocytic mitochondria in metabolic and amyloidogenic stress with implications of 17βHSD10 in multiple neurodegenerative mechanisms.

## Visual Abstract

**Figure:**
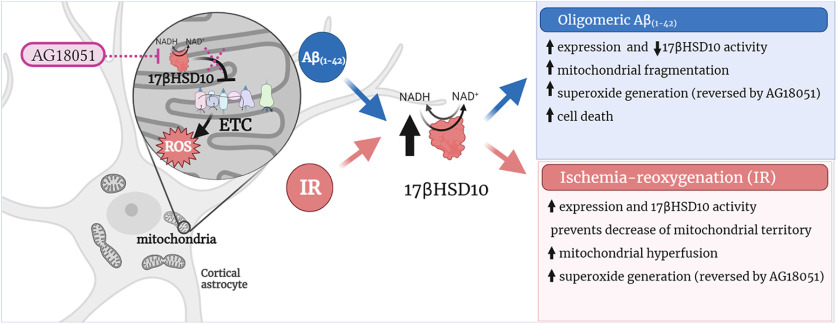

## Significance Statement

The current study presents the first direct evidence for the role of enzymatic activity of 17β-hydroxysteroid dehydrogenase type 10 (17βHSD10) in astrocytes. We report that the protein is involved in the response of cortical astrocytes to stress conditions associated with ischemic stroke and Alzheimer’s disease (AD). Furthermore, 17βHSD10 regulates astrocytic mitochondrial function, and the effects differ from what has been reported in neurons. These findings contribute to a growing body of evidence showing that astrocytic mitochondria are a key factor in neurodegenerative pathology. Considering the 17βHSD10-targeting therapeutics currently being developed for AD, these findings provide important insight into the role of this target in the cell population which carries out key metabolic support and toxic clearance from the brain.

## Introduction

Alzheimer’s disease (AD) and postischemic neurodegeneration are two of the main conditions causing dementia worldwide (Pluta et al., 2021). Clinical, epidemiological and experimental studies have shown that the two conditions may share the same or closely related pathologic mechanisms, including brain metabolic dysfunction (Kunz and Iadecola, 2008; Hammond et al., 2020), disruption of the blood-brain barrier (Zlokovic, 2008; Giraud et al., 2015), neuroinflammation and amyloid accumulation in the extracellular space (Jobst et al., 1994; Pluta et al., 1998; Grothe et al., 2018). Furthermore, AD increases the risk of stroke (Tolppanen et al., 2013) and vice versa (Ballard et al., 2000; Pluta and Ułamek, 2008). This association may be key in identifying the causative mechanisms driving neurodegeneration in these pathologies.

Although astrocytes are essential players in all of the aforementioned mechanisms (Guo et al., 2012; Chaitanya et al., 2014; Grubman et al., 2019; Habib et al., 2020), the role of their mitochondria has been mostly neglected because of the well-established observation that astrocytes are normally glycolysis-dependent (Pellerin and Magistretti, 1994; Magistretti and Pellerin, 1999; Magistretti, 2006). However, disease-associated metabolic stress marked by low levels of brain glucose (Mosconi, 2005; Oh et al., 2016) imposes metabolic load on astrocytic mitochondria to use alternative energy fuels to sustain the brain (Hoyer et al., 1991; Guzmán and Blázquez, 2004; Nilsen et al., 2007; Xu et al., 2016; Polyzos et al., 2019). Furthermore, immortalized hippocampal astrocytes from 3xTg-AD mice (3Tg-iAstro cells) have an impaired bioenergetics profile characterized by reduced glycolysis and mitochondrial respiration, accompanied by an increased generation of reactive oxygen species (ROS; Dematteis et al., 2020). Impairing mitochondrial respiration in astrocytes also increases astrogliosis, and neurodegeneration in a mouse stroke model (Fiebig et al., 2019). In this regard, mitochondrial bioenergetics and metabolic flexibility would be a key determinant in the survival and onset of neurodegeneration.

17β-hydroxysteroid dehydrogenase type 10 (17βHSD10) is a mitochondrial short-chain dehydrogenase/reductase (SDR; He et al., 1998) with a wide-ranging substrate specificity (Powell et al., 2000; He and Yang, 2006). It includes the catalysis of the third step of β-oxidation (Yang et al., 2005), catabolism of isoleucine (Luo et al., 1995), and neurosteroid metabolism of 17β-estradiol, allopregnanolone, and 3α,5α−3,21-dihydroxypregnan-20-one (Belelli and Lambert, 2005). Independent of its catalytic function, 17βHSD10 has a structural role in mitochondria as one of the three components of ribonuclease P complex which is essential for mitochondrial tRNA processing (Holzmann et al., 2008; Deutschmann et al., 2014). Missense mutations in the gene encoding 17βHSD10 cause progressive neurodegeneration which affects motor skills, speech, and vision in childhood (Zschocke, 2012).

17βHSD10 has also been known under the name of amyloid binding alcohol dehydrogenase (ABAD) because of its capacity to bind β-amyloid (Aβ; Lustbader et al., 2004; Yan et al., 2007), which is a key pathologic marker in AD (Querfurth and LaFerla, 2010). The complex was later proposed to obstruct the binding of 17βHSD10 to cyclophilin D, leading to increased formation of mitochondrial permeability pore (Yan and Stern, 2005). However, it is still unclear whether this is the sole mechanism through which 17βHSD10 affects AD pathology and whether it is only neurons that mediate these mechanisms.

He et al. (2005) proposed that 17βHSD10 is overexpressed in astrocytes surrounding amyloid plaques and proposed that astrocytic 17βHSD10 might be important for allopregnanolone metabolism in these cells. While there is no further confirmation of their immunohistological data, others have suggested that 17βHSD10 might not be detected in astrocytes (Fukuzaki et al., 2008). Nevertheless, a recent transcriptomic analysis of the adult mouse brain also shows that 17βHSD10 levels are significantly higher in astrocytes as compared with neurons (Saunders et al., 2018). Although these initial studies proposed that astrocytic 17βHSD10 may be important for normal and disease physiology, the non-neuronal role of the protein remains elusive.

The current study addresses this lack in literature and offers direct evidence that the protein is involved in the astrocytic response to metabolic and amyloidogenic stress. To isolate astrocyte-specific events in the absence of other cell types, we examined the structural and enzymatic role of 17βHSD10 in an *in vitro* culture model of murine astrocytes. We first established that the enzyme is expressed and active at comparable levels in cortical, hippocampal, and cerebellar astrocytes. Furthermore, we found that the expression and enzymatic activity of 17βHSD10 are affected by amyloidogenic and ischemic stress. In turn, the overexpression of catalytically active (as opposed to inhibited or mutated and inactive) 17βHSD10 inhibits respiration and has multiple implications for mitochondrial function and astrocytic response to ischemia and Aβ treatment.

## Materials and Methods

### Primary culture and tissue slice preparation

The current methodology was designed and performed in accordance with the United Kingdom Animals (Scientific Procedures) Act 1986 and was approved by the University of St Andrews Animal Welfare and Ethics Committee.

Astrocytic cultures were obtained from postnatal day (p)1–p2 neonatal male and female C57BL/6J mice similar to a detailed procedure published by Qiu et al. (2018). The male:female ratio was not assessed in every litter, however the average ratio for the breeders was 4:3 males to females. Briefly, mouse brains were harvested and subdissected to isolate cortical, hippocampal, and cerebellar tissue. Meninges were removed, and the three brain regions were diced with a sterile surgical blade. Each sample was then enzymatically digested in 2 mL papain solution [81.8 mm Na_2_SO_4_ (Sigma #S5640), 30.01 mm K_2_SO_4_ (Sigma #P9458), 5.84 mm MgCl_2_ (Sigma #M8266), CaCl_2_ 0.25 mm (Sigma #C1016), 2 mm HEPES (Sigma #H0887), 0.001% phenol red (Sigma #P0290), 18 mm D-glucose solution (Sigma #G8769), 3.71 mm L-cysteine (Sigma #168149), 1 mm kynurenic acid (Sigma #K3375), and 10 U/mL papain (Sigma #P4762)] at 37°C for 40 min, replacing the solution halfway and periodically shaking the sample. The digested tissue was then washed four times with astrocytic growth medium [DMEM (ThermoFisher, #A1443001) supplemented with 5.5 mm glucose, 45% D-glucose (Sigma, #G8769), 2 mm L-glutamine (ThermoFisher, #G7513), 1 mm sodium pyruvate (Sigma, #S8636), 1% PenStrep (Invitrogen, #15140122), and 10% fetal bovine serum (Invitrogen, #26140079)]. The tissue was dissociated in fresh growth medium and plated into poly-D-lysine (PDL) precoated T75 flasks.

The culture medium was replaced every 48 h, which was always preceded by a gentle wash with PBS to remove loosely attached populations of microglia and oligodendrocytes (Lange et al., 2012). After a week, *in vitro* cultures were briefly washed with PBS+TrypLE (5%) to further facilitate the removal of non-astrocytic populations. The cultures were then dissociated with 2 mL TrypLE (ThermoFisher, #12604-039) for 8 min at 37°C and seeded into appropriate cell culture vessels precoated with PDL at a density of 15,000 cells per cm^2^. The resulting cell populations had comparable purity levels as indicated by the traditional astrocytic marker GFAP (glial fibrillary acidic protein) and the pan-astrocytic marker aldehyde dehydrogenase 1 family, member L1 (ALDH1L1; Cahoy et al., 2008) with preparations comprising over 98% ALDH1L1^+^ and over 85% GFAP^+^ cells.

Tissue slices were prepared by humanely killing neonatal and adult male mice, isolating and slicing the brain using McIlwain tissue chopper to generate 350-μm-thick brain slices. The slices subsequently placed in 4% paraformaldehyde (PFA) and fixed overnight at 4°C.

### Cell line culturing

The human embryonic kidney 293T/17 (HEK293T/17) cell line used for lentiviral packaging was obtained from ATCC. These cells were maintained in DMEM [(ThermoFisher, #31966021), supplemented with 10% fetal bovine serum (Invitrogen, #26140079) and 1% PenStrep (Invitrogen, #15140122)] at 37°C, 5% CO_2_, humidified atmosphere. The growth medium was replaced every 3 d and cultures were passaged at 80% confluency using TrypLE (ThermoFisher, #12604-039).

### Molecular cloning and viral transduction

The lentiviral plasmids in this work were created using donor pcDNA3 plasmid containing human 17βHSD10 with attached mitochondrial targeting sequence (MTS). The wild-type (wt) pcDNA3-MTS-17βHSD10 and the mutated variant pcDNA3-MTS-17βHSD10-Y168G were kindly provided by Dr. Patrick Guest and Dr. Madhurima Dey. The catalytically inactive form of 17βHSD10 was a kind gift from Dr. Patrick Guest who generated the mutant through site-directed mutagenesis of tyrosine (Y168; Guest, 2016), an amino acid which is part of the highly conserved catalytic triad of the enzyme (S155, Y168, K172; Yan et al., 1999; Kissinger et al., 2004). Tyrosine was replaced by glycine (Y168G), as previously done by Yan et al. (1999). The 17βHSD10 sequence used is as follows:

ATGGCAGCAGCGTGTCGGAGCGTGAAGGGCCTGGTGGCGGTAATAACCGGAGGAGCCTCGGGCCTGGGCCTGGCCACGGCGGAGCGACTTGTGGGGCAGGGAGCCTCTGCTGTGCTTCTGGACCTGCCCAACTCGGGTGGGGAGGCCCAAGCCAAGAAGTTAGGAAACAACTGCGTTTTCGCCCCAGCCGACGTGACCTCTGAGAAGGATGTGCAAACAGCTCTGGCTCTAGCAAAAGGAAAGTTTGGCCGTGTGGATGTAGCTGTCAACTGTGCAGGCATCGCGGTGGCTAGCAAGACGTACAACTTAAAGAAGGGCCAGACCCATACCTTGGAAGACTTCCAGCGAGTTCTTGATGTGAATCTCATGGGCACCTTCAATGTGATCCGCCTGGTGGCTGGTGAGATGGGCCAGAATGAACCAGACCAGGGAGGCCAACGTGGGGTCATCATCAACACTGCCAGTGTGGCTGCCTTCGAGGGTCAGGTTGGACAAGCTGCATACTCTGCTTCCAAGGGGGGAATAGTGGGCATGACACTGCCCATTGCTCGGGATCTGGCTCCCATAGGTATCCGGGTGATGACCATTGCCCCAGGTCTGTTTGGCACCCCACTGCTGACCAGCCTCCCAGAGAAAGTGTGCAACTTCTTGGCCAGCCAAGTGCCCTTCCCTAGCCGACTGGGTGACCCTGCTGAGTATGCTCACCTCGTACAGGCCATCATCGAGAACCCATTCCTCAATGGAGAGGTCATCCGGCTGGATGGGGCCATTCGTATGCAGCCTTGA, where TA (underlined) is replaced by GG in the Y168G mutated version.

The desired sequences were PCR-amplified out of the pcDNA3 vector while 5′ XbaI and 3′ SalI restriction sites were added. The newly generated donor sequences as well as the recipient lentiviral construct pLenti-CMV-GFP-Puro (658-5; Addgene, #17448, a gift from Eric Campeau and Paul Kaufman) were digested with restriction enzymes XbaI and SalI (NEB). The GFP sequence was removed and the desired MTS-17βHSD10/Y168G were inserted instead. One Shot^TM^ Stbl3^TM^ competent *Escherichia coli* (ThermoFisher, #C737303) were used to produce and isolate the newly generated pLenti-CMV-MTS-17βHSD10/Y168G-Puro. QIAGEN Miniprep (QIAGEN, #27104) and Maxiprep (QIAGEN, #12163) kits were used to purify DNA from overnight liquid bacterial cultures following manufacturer’s instructions.

The newly generated lentiviral constructs were transduced into primary astrocytes using the packaging psPAX2 (Addgene, #12260) plasmid, and the envelope pMD2.G-VSV.G (Addgene, #8454) plasmid. Briefly, the three second generation plasmids (packaging psPAX2, envelope pMD2.G-VSV.G and pLenti-CMV-MTS-17βHSD10-Puro or pLenti-CMV-MTS-17βHSD10-Y168G-Puro) were incubated with TransIT-LT1 Transfection reagent in a 3:1 ratio (μl reagent: μg DNA) in 1.5 mL OptiMEM (ThermoFisher, #31985062). The mix was transfected into HEK293T cells for 16 h. The lentiviral particles were collected over the following 48 h. The viral particles were then filtered through a 0.45-μm filter unit (Sigma, #SLHV004SL), diluted into astrocyte culture medium and applied to primary astrocytes. After 4 h, the transduction mix was removed, and astrocytes were cultured for five more days before experimental treatment.

**Table 1:** Statistical table

Figure	Test	*p*-value	Shapiro–Wilk	Sample	*N*	Mean	SEM
Figure 1*C*	One-way ANOVA with Tukey’s *post hoc* test	0.452	0.126	17βHSD10-cortex	5	1.094	0.096
			0.949	17βHSD10 hippocampus	5	1.084	0.060
			0.858	17βHSD10-cerebellum	5	1.221	0.089
	One-way ANOVA with Tukey’s *post hoc* test	0.420	<0.001	COXIV-cortex	5	1.096	0.091
			0.992	COXIV-hippocampus	5	1.002	0.084
			0.098	COXIV-cerebellum	5	1.153	0.059
	One-way ANOVA with Tukey’s *post hoc* test	0.760	0.833	17βHSD10/COXIV-cortex	5	1.061	0.137
			0.454	17βHSD10/COXIV-hippocampus	5	1.203	0.100
			0.311	17βHSD10/COXIV-cerebellum	5	1.143	0.160
Figure 1*E*	Mixed ANOVA		0.849	cortex-0 min	6	4972.333	26.911
	Time (main)	<0.001	0.049	cortex-10 min	6	5023.167	34.622
	Region (main)	0.583	0.610	cortex-20 min	6	5173.000	37.605
	interaction	0.170	0.159	cortex-30 min	6	5297.000	50.440
			0.584	hippocampus-0 min	6	4974.333	26.911
			0.096	hippocampus-10 min	6	5007.167	34.622
			0.407	hippocampus-20 min	6	5127.500	37.605
			0.059	hippocampus-30 min	6	5186.333	50.440
			0.119	cerebellum-0 min	6	4969.167	26.911
			0.332	cerebellum-10 min	6	5069.000	34.622
			0.552	cerebellum-20 min	6	5176.500	37.605
			0.562	cerebellum-30 min	6	5252.167	50.440
Figure 2*A*	One-way ANOVA with Tukey’s *post hoc* test	<0.001	0.042	control	7	1.191	0.316
			0.258	mut-17βHSD10	7	18.429	2.879
			0.078	wt-17βHSD10	7	18.573	3.096
Figure 2*B*	One-way ANOVA with Tukey’s *post hoc* test	<0.001	0.244	control	9	113.119	1.098
			0.781	mut-17βHSD10	9	115.079	1.570
			0.018	wt-17βHSD10	9	439.268	89.354
Figure 2*D*	Mixed linear model with Tukey’s *post hoc* test		<0.001	control	254	685.867	17.373
			<0.001	mut-17βHSD10	244	703.992	20.494
			<0.001	wt-17βHSD10	254	684.278	15.635
	Main effect of expression	*F*_(2,743)_ = 0.308, *p* = 0.691, η_p_^2^ = 0.133					
	Main effect of culture replicate	*F*_(2,743)_ = 0.029, *p* = 0.971, η_p_^2^ = 0.014					
	Interaction	*F*_(4,743)_ = 1.331, *p* = 0.257, η_p_^2^ = 0.007					
Figure 2*E*	Mixed linear model with Tukey’s *post hoc* test		<0.001	control	255	1.761	0.021
			<0.001	mut-17βHSD10	235	2.049	0.033
			<0.001	wt-17βHSD10	223	2.002	0.030
	Main effect of expression	*F*_(2,704)_ = 30.155, *p* = 0.004, η_p_^2^ = 0.938					
	Main effect of culture replicate	*F*_(2,704)_ = 0.211, *p* = 0.818, η_p_^2^ = 0.095					
	Interaction	*F*_(4,704)_ = 1.075, *p* = 0.368, η_p_^2^ = 0.006					
Figure 2*F*	Mixed linear model with Tukey’s *post hoc* test	<0.001	0.001	control	253	2.929	0.058
			<0.001	mut-17βHSD10	246	4.172	0.078
			<0.001	wt-17βHSD10	225	3.971	0.079
	Main effect of expression	*F*_(2,715)_ = 212.558, *p* < 0.001, η_p_^2^ = 0.991					
	Main effect of culture replicate	*F*_(2,715)_ = 0.449, *p* = 0.667, η_p_^2^ = 0.182					
	Interaction	*F*_(4,715)_ = 0.417, *p* = 0.797, η_p_^2^ = 0.002					
Figure 2*G*	Mixed linear model with Tukey’s *post hoc* test	0.009	<0.001	control	255	2.677	0.095
			<0.001	mut-17βHSD10	246	3.070	0.111
			<0.001	wt-17βHSD10	203	3.078	0.119
	Main effect of expression	*F*_(2,715)_ = 8.267, *p* = 0.256, η_p_^2^ = 0.494					
	Main effect of culture replicate	*F*_(2,715)_ = 0.254, *p* = 0.787, η_p_^2^ = 0.112					
	Interaction	*F*_(4,715)_ = 2.535, *p* = 0.0.39, η_p_^2^ = 0.014					
Figure 3*B*	One-way ANOVA with Tukey’s *post hoc* test	0.124		Basal respiration			
			0.196	control	11	9459.894	702.399
			0.319	mut-17βHSD10	11	9365.145	461.574
			0.207	wt-17βHSD10	11	8227.585	634.004
			0.525	wt-17βHSD10-AG18051	11	10,289.873	550.445
	One-way ANOVA with Tukey’s *post hoc* test	<0.001		Maximal respiration			
			0.524	control	11	16,182.764	849.316
			0.566	mut-17βHSD10	11	15,564.403	692.724
			0.663	wt-17βHSD10	11	10,644.094	931.124
			0.749	wt-17βHSD10-AG18051	11	17,685.873	740.995
	One-way ANOVA with Tukey’s *post hoc* test	<0.001		Spare capacity			
			0.471	control	11	6722.869	287.169
			0.854	mut-17βHSD10	11	6199.257	407.046
			0.675	wt-17βHSD10	11	2416.509	485.998
			0.902	wt-17βHSD10-AG18051	11	7396.000	511.162
	One-way ANOVA with Tukey’s *post hoc* test	<0.001		ATP			
			0.205	control	11	7116.955	510.235
			0.987	mut-17βHSD10	11	6894.442	377.057
			0.295	wt-17βHSD10	11	6103.095	494.693
			0.398	wt-17βHSD10-AG18051	11	7620.969	426.466
	One-way ANOVA with Tukey’s *post hoc* test	0.248		Proton leak			
			0.322	control	11	2342.939	218.751
			0.047	mut-17βHSD10	11	2470.702	236.700
			0.026	wt-17βHSD10	11	2124.490	171.068
			0.210	wt-17βHSD10-AG18051	11	2767.481	261.637
Figure 3*C*	One-way ANOVA with Tukey’s *post hoc* test	<0.001		mut-17βHSD10			
			0.680	control	6	105.412	2.603
			0.268	FCCP	6	99.763	3.576
			0.868	OM	6	100.262	9.700
			0.385	AA	6	69.928	3.920
			0.563	ROT	6	63.441	3.079
	One-way ANOVA with Tukey’s *post hoc* test	0.239		mut-17βHSD10-AG18051			
			0.601	control	5	96.463	7.560
			0.576	FCCP	5	97.961	6.000
			0.523	OM	5	86.966	7.697
			0.046	AA	5	98.821	10.812
			0.213	ROT	5	77.290	3.938
	One-way ANOVA with Tukey’s *post hoc* test	<0.001		wt-17βHSD10			
			0.219	control	6	174.683	3.501
			0.079	FCCP	6	197.215	7.434
			0.435	OM	6	185.665	28.768
			0.158	AA	6	114.067	7.088
			0.858	ROT	6	87.097	4.562
	One-way ANOVA with Tukey’s *post hoc* test	0.168		wt-17βHSD10-AG18051			
			0.827	control	4	99.756	10.407
			0.745	FCCP	5	102.113	4.069
			0.552	OM	5	92.224	6.437
			0.126	AA	5	102.260	10.680
			0.086	ROT	5	78.412	5.266
Figure 3*D*	One-way ANOVA with Tukey’s *post hoc* test	<0.001	0.205	control	11	174.205	5.353
			0.897	mut-17βHSD10	11	167.062	5.353
			0.495	wt-17βHSD10	11	129.654	5.353
			0.451	wt-17βHSD10-AG18051	7	166.592	6.710
Extended Data Fig. 3-1*B*	Two-tailed unpaired *t* tests	0.337		Basal respiration Control AG18051	11 6	9460 10,712	702.4 1137
		0.036		Maximal respiration Control AG18051	11 6	16,183 19,850	849.3 1516
		0.005		Spare capacity Control AG18051	11 6	6723 9138	287.2 868.8
		0.741		ATP production Control AG18051	11 6	7117 7435	510.2 875.7
		0.023		Proton leak Control AG18051	11 6	2343 3372	218.8 381.3
Figure 4*B*	One-way ANOVA with Tukey’s *post hoc* test	0.005		17βHSD10			
			0.898	Control	6	0.925	0.133
			0.066	IR	6	1.431	0.121
			0.176	IR+glucose	6	0.852	0.083
	One-way ANOVA with Tukey’s *post hoc* test	<0.001		VDAC1			
			0.873	Control	6	0.850	0.089
			0.592	IR	6	1.840	0.172
			0.203	IR+glucose	6	0.891	0.110
	One-way ANOVA with Tukey’s *post hoc* test	0.442		17βHSD10/COXIV			
			0.753	Control	5	0.864	0.099
			0.005	IR	5	0.766	0.098
			0.055	IR+glucose	5	0.693	0.076
Figure 4*C*	Mixed ANOVA		0.314	control-control	12	100.000	3.410
	Group (main)	0.290	0.112	control-IR	12	82.999	4.985
	Treatment (main)	<0.001	0.716	mut-17βHSD10-control	12	103.521	4.272
	interaction	0.977	0.533	mut-17βHSD10-IR	12	86.108	2.443
			0.145	wt-17βHSD10-control	12	101.045	3.106
			0.271	wt-17βHSD10-IR	12	85.337	5.805
Figure 4*D*	Mixed ANOVA		0.026	control-control	12	2.221	0.243
	Group (main)	0.002	0.020	control-IR	12	15.781	3.006
	Treatment (main)	0.522	0.050	mut-17βHSD10-control	12	1.563	0.212
	interaction	<0.001	0.189	mut-17βHSD10-IR	12	15.269	3.001
			0.500	wt-17βHSD10-control	12	2.043	0.116
			0.088	wt-17βHSD10-IR	12	16.045	2.333
Figure 5*A*	Mixed ANOVA						
	Group (main)	<0.001	0.244	control-control	6	127.860	9.436
	Treatment (main)	<0.001	0.901	control-IR	6	323.533	4.313
	interaction	<0.001	0.582	control-IR-AG18051	6	173.563	4.331
			0.141	mut-17βHSD10-control	6	140.961	9.189
			0.114	mut-17βHSD10-IR	6	283.245	7.154
			0.031	mut-17βHSD10-IR-AG18051	6	215.737	22.617
			0.655	wt-17βHSD10-control	6	361.384	26.606
			0.011	wt-17βHSD10-IR	6	1026.559	133.923
			0.070	wt-17βHSD10-IR-AG18051	6	196.246	18.773
Figure 5*B*	Mixed ANOVA		0.475	control-control	6	96.693	6.355
	Group (main)	0.093	0.029	control-IR	3	160.736	8.422
	Treatment (main)	<0.001	0.830	control-IR-AG18051	3	112.557	21.545
	interaction	0.029	0.385	mut-17βHSD10-control	6	96.662	16.852
			0.322	mut-17βHSD10-IR	3	150.052	36.172
			0.179	mut-17βHSD10-IR-AG18051	3	137.171	21.279
			0.153	wt-17βHSD10-control	6	85.644	9.051
			0.776	wt-17βHSD10-IR	3	240.813	12.960
			0.673	wt-17βHSD10-IR-AG18051	3	133.154	18.287
Figure 5*C*	Two-way ANOVA			control-control	7	2.879	0.393
	Group (main)	<0.001		control-IR	7	8.036	0.840
	Treatment (main)	0.444		control-IR-AG18051	7	8.943	1.399
	interaction	<0.001		mut-17βHSD10-control	21	2.658	0.503
				mut-17βHSD10-IR	21	8.764	0.960
				mut-17βHSD10-IR-AG18051	21	6.591	1.647
				wt-17βHSD10-control	12	1.875	0.369
				wt-17βHSD10-IR	12	11.429	0.834
				wt-17βHSD10-IR-AG18051	12	2.905	0.495
Figure 5*E*	Mixed linear model with Tukey’s *post hoc* test		<0.001	control-control	45	640.292	34.194
			<0.006	control-IR	45	254.570	15.413
			0.022	mut-17βHSD10-control	45	719.772	35.778
			0.039	mut-17βHSD10-IR	45	647.206	35.228
			0.012	wt-17βHSD10-control	45	694.020	34.624
			0.051	wt-17βHSD10-IR	45	903.350	39.936
	Main effect of expression	*F*_(1,243)_ = 22.702, *p* = 0.006
	Main effect of treatment	*F*_(1,243)_ = 38.381, *p* = 0.022
	Interaction	*F*_(2,243)_ = 96.796, *p* < 0.001
	Main effect of culture replicate	*F*_(2,243)_ = 0.674, *p* = 0.568
Figure 5*F*	Mixed linear model with Tukey’s *post hoc* test		<0.001	control-control	45	1.420	0.025
			0.331	control-IR	45	1.394	0.017
			0.941	mut-17βHSD10-control	45	1.633	0.012
			0.412	mut-17βHSD10-IR	45	1.542	0.024
			0.126	wt-17βHSD10-control	45	1.774	0.025
			<0.001	wt-17βHSD10-IR	45	1.685	0.016
	Main effect of expression	*F*_(1,243)_ = 14.491, *p* = 0.015
	Main effect of treatment	*F*_(1,243)_ = 15.029, *p* = 0.060
	Interaction	*F*_(2,243)_ = 0.190, *p* = 0.834
	Main effect of culture replicate	*F*_(2,243)_ = 0.399, *p* = 0.941
Figure 5*G*	Mixed linear model with Tukey’s *post hoc* test		0.018	control-control	45	1.949	0.074
			0.308	control-IR	45	1.563	0.065
			0.046	mut-17βHSD10-control	45	4.152	0.144
			0.003	mut-17βHSD10-IR	45	2.661	0.158
			0.005	wt-17βHSD10-control	45	4.350	0.204
			0.128	wt-17βHSD10-IR	45	4.501	0.121
	Main effect of expression	*F*_(1,243)_ = 65.562, *p* = 0.001					
	Main effect of treatment	*F*_(1,243)_ = 8.388, *p* = 0.101					
	Interaction	*F*_(2,243)_ = 7.770, *p* = 0.041					
	Main effect of culture replicate	*F*_(2,243)_ = 0.612, *p* = 0.629					
Figure 5*H*	Mixed linear model with Tukey’s *post hoc* test		0.689	control-control	45	1.352	0.036
			0.772	control-IR	45	1.309	0.025
			0.062	mut-17βHSD10-control	45	2.530	0.077
			0.003	mut-17βHSD10-IR	45	1.684	0.081
			<0.001	wt-17βHSD10-control	45	2.443	0.108
			0.158	wt-17βHSD10-IR	45	2.682	0.076
	Main effect of expression	*F*_(1,243)_ = 45.037, *p* = 0.002					
	Main effect of treatment	*F*_(1,243)_ = 6.758, *p* = 0.121					
	Interaction	*F*_(2,243)_ = 22.136, *p* = 0.007					
	Main effect of culture replicate	*F*_(2,243)_ = 0.232, *p* = 0.805					
	One-way ANOVA with Tukey’s *post hoc* test	0.019		17βHSD10			
Figure 6*B*			0.900	0 nm	5	0.972	0.113
			0.650	100 nm	5	1.317	0.081
			0.819	1000 nm	5	1.366	0.072
	One-way ANOVA with Tukey’s *post hoc* test	0.809		VDAC1			
			<0.001	0 nm	3	1.067	0.067
			0.949	100 nm	3	1.029	0.141
			0.902	1000 nm	3	1.155	0.182
	One-way ANOVA with Tukey’s *post hoc* test	0.528		17βHSD10/COXIV			
			0.463	0 nm	5	0.944	0.067
			0.683	100 nm	5	0.775	0.061
			0.202	1000 nm	5	0.851	0.154
Figure 6*C*	Mixed ANOVA		0.304	control-control	8	109.015	4.408
	Group (main)	0.002	0.815	control-Aβ	8	115.155	5.896
	Treatment (main)	0.165	0.209	mut-17βHSD10-control	8	112.757	5.435
	interaction	0.044	0.229	mut-17βHSD10-Aβ	8	109.097	4.470
			0.508	wt-17βHSD10-control	8	104.241	6.616
			0.034	wt-17βHSD10-Aβ	8	83.870	3.612
Figure 6*D*	Mixed ANOVA		0.216	control-control	8	0.873	0.385
	Group (main)	0.005	0.003	control-Aβ	8	3.202	0.397
	Treatment (main)	<0.001	0.395	mut-17βHSD10-control	8	0.942	0.334
	interaction	0.285	0.761	mut-17βHSD10-Aβ	8	3.248	0.375
			0.698	wt-17βHSD10-control	8	1.485	0.302
			0.076	wt-17βHSD10-Aβ	8	4.815	0.379
Figure 7*A*	Mixed ANOVA						
	Group (main)	<0.001	0.796	control-control	8	97.837	3.182
	Treatment (main)	<0.001	0.870	control-Aβ	8	98.334	2.863
	interaction	<0.001	0.454	control-Aβ-AG18051	8	104.191	3.936
			0.147	mut-17βHSD10-control	8	96.317	2.262
			0.827	mut-17βHSD10-Aβ	8	91.433	4.324
			0.859	mut-17βHSD10-Aβ-AG18051	8	95.197	4.067
			0.713	wt-17βHSD10-control	8	374.482	11.633
			0.444	wt-17βHSD10-Aβ	8	324.850	11.826
			0.168	wt-17βHSD10-Aβ-AG18051	8	104.093	2.924
Figure 7*B*	Mixed ANOVA		0.641	control-control	8	100.000	10.408
	Group (main)	0.242	0.400	control-Aβ	8	171.792	24.816
	Treatment (main)	<0.001	0.901	control-Aβ-AG18051	8	103.411	16.303
	interaction	0.204	0.108	mut-17βHSD10-control	8	106.125	10.044
			0.150	mut-17βHSD10-Aβ	8	172.123	22.324
			0.788	mut-17βHSD10-Aβ-AG18051	8	78.465	16.038
			0.003	wt-17βHSD10-control	8	114.667	13.606
			0.296	wt-17βHSD10-Aβ	8	241.006	39.597
			0.111	wt-17βHSD10-Aβ-AG18051	8	81.276	7.345
Figure 7*C*	Two-way ANOVA			control-control	8	0.873	0.385
				control-Aβ	8	4.024	0.182
	Group (main)	0.017		control-Aβ-AG18051	8	3.558	0.589
	Treatment (main)	<0.001		mut-17βHSD10-control	8	0.942	0.333
	interaction	0.521		mut-17βHSD10-Aβ	8	3.274	0.375
				mut-17βHSD10-Aβ-AG18051	8	2.590	0.413
			wt-17βHSD10-control	8	1.784	0.266
			wt-17βHSD10-Aβ	8	4.606	0.447
			wt-17βHSD10-Aβ-AG18051	8	3.284	0.474
Figure 7*E*	Mixed linear model with Tukey’s *post hoc* test		0.048	control-control	120	738.426	26.021
			0.006	control-Aβ	120	559.937	22.975
			<0.001	mut-17βHSD10-control	120	796.907	32.100
			<0.001	mut-17βHSD10-Aβ	120	536.263	25.395
			<0.001	wt-17βHSD10-control	120	743.826	22.845
			0.231	wt-17βHSD10-Aβ	120	476.186	15.730
	Main effect of expression	*F*_(2,702)_ = 0.500, *p* = 0.640					
	Main effect of treatment	*F*_(1,702)_ = 30.749, *p* = 0.031					
	Interaction	*F*_(2,702)_ = 0.668, *p* = 0.562					
	Main effect of culture replicate	*F*_(2,702)_ = 1.113, *p* = 0.454					
Figure 7*F*	Mixed linear model with Tukey’s *post hoc* test		0.006	control-control	120	2.004	0.024
			<0.001	control-Aβ	120	1.804	0.034
			0.007	mut-17βHSD10-control	120	2.439	0.036
			<0.001	mut-17βHSD10-Aβ	120	1.933	0.040
			<0.001	wt-17βHSD10-control	120	2.324	0.034
			<0.001	wt-17βHSD10-Aβ	120	1.681	0.013
	Main effect of expression	*F*_(2,702)_ = 3.629, *p* = 0.034					
	Main effect of treatment	*F*_(1,702)_ = 78.927, *p* = 0.012					
	Interaction	*F*_(2,702)_ = 21.179, *p* = 0.007					
	Main effect of culture replicate	*F*_(2,702)_ = 2.323, *p* = 0.226					
Figure 7*G*	Mixed linear model		0.011	control-control	120	3.194	0.084
		<0.001	0.399	control-Aβ	120	3.127	0.081
		<0.001	<0.001	mut-17βHSD10-control	120	4.382	0.117
		<0.001	0.066	mut-17βHSD10-Aβ	120	4.463	0.111
			<0.001	wt-17βHSD10-control	120	4.080	0.110
			<0.001	wt-17βHSD10-Aβ	120	2.753	0.150
	Main effect of expression	*F*_(2,702)_ = 5.364, *p* = 0.074					
	Main effect of treatment	*F*_(1,702)_ = 22.354, *p* = 0.042					
	Interaction	*F*_(2,702)_ = 12.575, *p* = 0.019					
	Main effect of culture replicate	*F*_(2,702)_ = 0.526, *p* = 0.640					
Figure 7*H*	Mixed linear model		<0.001	control-control	120	3.311	0.127
			<0.001	control-Aβ	120	3.148	0.110
			<0.001	mut-17βHSD10-control	120	3.709	0.201
			<0.001	mut-17βHSD10-Aβ	120	4.070	0.187
			<0.001	wt-17βHSD10-control	120	4.130	0.189
			<0.001	wt-17βHSD10-Aβ	120	3.111	0.189
	Main effect of expression	*F*_(2,702)_ = 2.047, *p* = 0.244
	Main effect of treatment	*F*_(1,702)_ = 207.886, *p* = 0.005
	Interaction	*F*_(2,702)_ = 13.591, *p* = 0.016					
	Main effect of culture replicate	*F*_(2,702)_ = 3.066, *p* = 0.216					

### Viability assays

#### Alamar blue

Viability of primary astrocytes was assessed via an alamar blue assay (ThermoFisher, #DAL1025). Cells were seeded into PDL-coated black wall clear bottom 96-well plates (Greiner, #655090). Following experimental treatment, alamar blue was added (10% v/v) and incubated for 4 h at 37°C. Resazurin, the active compound of the assay, is metabolized to resorufin in living cells. Resazurin fluorescence was detected using SpectraMaxM2e spectrophotometer (Molecular Devices) using 570-nm excitation and 590-nm detection wavelengths. The signal was corrected for background fluorescence and the viability was calculated as a % control (untreated) cells.

#### Lactate dehydrogenase (LDH) assay

Cytotoxicity was determined using LDH assay (ThermoFisher, #88953), a colorimetric assay detecting LDH release into culture medium following cell membrane rupture. Following manufacturer’s instructions, 50% (v/v) LDH reaction mix was added to cell medium removed from the treated astrocytes. Following a 30 min incubation at room temperature, LDH concentration was measured by detecting absorbance at 490 and 680 nm using a SpectraMaxM2e spectrophotometer (Molecular Devices). The % cytotoxicity was calculated via the following formula:

$$\%cytotoxicity=\frac{LDH_{treatment}-LDH_{spont}}{LDH_{max}-LDH_{spont}}\times 100.$$

LDH_treatment_ indicates LDH activity measured in astrocytes exposed to different treatments, LDH_spont_ is the level of activity in water-treated control; LDH_max_ is the maximum LDH activity in medium where cells were lysed with 10× lysis buffer (supplied with the kit).

### Immunocytochemistry and immunohistochemistry

Mitochondria were stained with MitoTracker Red CMXRos (ThermoFisher, #M7512). The reagent was prepared as 1 mm stock solution in DMSO and further diluted to 100 nm working concentration in serum-free, phenol red-free astrocyte culture medium. The staining solution was incubated with astrocytes grown on PDL-coated glass coverslips (VWR, #631-0150P) for 20 min at 37°C.

For immunocytochemistry, the cells were then fixed with 4% PFA (Pierce, #28906) or ice-cold methanol (VWR, # 20847.240). Following blocking and permeabilization in PBS containing 10% goat serum (Sigma, #G9023) and 0.4% Triton X-100 for 90 min, the primary antibodies [anti-ERAB/17βHSD10 (Abcam, #ab167410) 1:200; anti- ALDH1L1 (Abcam, #190298) 1:1000; anti-GFAP (Sigma, #G3893) 1:500] were diluted in blocking solution and incubated with the samples overnight at 4°C. The secondary antibodies [anti-mouse Alexa Fluor 488 (Invitrogen, #A-11001) 1:1000; anti-rabbit Alexa Fluor 568 (Invitrogen, #A-11011) 1:1000] were diluted in PBS and incubated with the samples for 60 min at room temperature. The coverslips were then washed in PBS and mounted on slides using Fluoroshield mounting medium (Abcam #ab104135), sealed with nail polish and stored at 4°C until imaging with Leica SP8 scanning confocal microscope.

Similarly, fixed brain slices were washed with PBS after overnight fixation in 4% PFA. This was followed by permeabilization and blocking in 0.4% Triton X-100 for 48 h at 4°C. Primary antibodies were diluted in blocking solution and incubated with the slices for 24 h at 4°C [anti-ERAB/17βHSD10 (Abcam, #ab137455) 1:500; anti-GFAP (Sigma, #G3893) 1:500]. The secondary antibodies [anti-mouse Alexa Fluor 488 (Invitrogen, #A-11001) 1:1000; anti-rabbit Alexa Fluor 647 (Invitrogen, #A-21245) 1:1000] were diluted in PBS and incubated with the samples for 2 h at room temperature. The slices were then washed with PBS, mounted on slices using Fluoroshield, sealed with nail polish, and stored at 4°C until imaging.

### Mitochondrial superoxide detection

Mitochondrial superoxide generation in astrocytes was determined using MitoSOX Red Mitochondrial Superoxide Indicator (ThermoFisher, #M36008). The reagent allows for a highly selective detection of mitochondrial superoxide in live cells, whereby the oxidation of a cationic derivative of dihydroethidum by superoxide generates a highly fluorescent product. The reagent was stored as a 5 mm stock in DMSO, which was diluted further to a 5 μm working solution in serum-free, phenol red-free astrocyte culture medium. The cells were incubated with the working solution for 5 min, washed with PBS and fluorescence was measured using FLUOstar Optima microplate reader (BMG; excitation at 510 nm and emission at 620 nm). Background fluorescence was subtracted, and superoxide generation was calculated as a % control by normalizing to the fluorescence signal recorded in control cells. H_2_O_2_-treated cells were used as a positive control confirming MitoSOX reliably detected oxidative stress generation in primary astrocytes.

### Western blot analysis

Cell lysates were prepared by centrifuging primary astrocytes (1000 × *g* for 10 min) following incubation with TrypLE. The pellets were lysed using RIPA buffer (50 mm Tris-HCl, pH 8.0, 150 mm NaCl, 0.1% Triton X-100, 0.5% sodium deoxycholate, 0.1% SDS, 1 mm sodium orthovanadate, 1 mm NaF) with protease inhibitor cocktail (Roche, #04693159001) for 30 min on ice. Samples were centrifuged at 13,000 × *g* for 15 min at 4°C. The supernatant was collected, protein concentration was measured using BCA assay (ThermoFisher, #23225) following manufacturer’s instructions.

Polyacrylamide gels were cast in Mini-PROTEAN Tetra Handcast system (Bio-Rad, #1658000FC). The samples were prepared by adding 5× Laemmli buffer (10% SDS, 50% glycerol, 25% 2-mercaptoethanol, and 0.315 m Tris-HCl, pH 6.8) to cell lysates and heating at 95°C for 10 min. After loading onto the polyacrylamide gels, the proteins were separated by electrophoresis at 120 V for 90 min (running buffer: 25 mm Tris-Base, 190 mm glycine, and 0.1% SDS, pH 8.3). Proteins were then transferred to a polyvinylidene difluoride (PVDF) membrane (Millipore, #IPVH00010) in a XCell II blot module (ThermoFisher, EI9051) at 25 V for 90 min using ice-cold transfer buffer [25 mm Tris-HCl, 190 mm glycine, and 20% (v/v) methanol, pH 8.3]. The membranes were blocked for 60 min in 5% (w/v) dried nonfat milk (Marvel) in TBS-T (50 mm Tris-HCl, 138 mm NaCl, 2.7 mm KCl, pH 7.4, and 0.1% Tween 20). Primary antibodies were diluted in blocking solution [anti-VDAC1 (Abcam, #ab14734) 1:1000; anti-ERAB/17βHSD10 (Abcam, #ab167410) 1:10,000; anti-COXIV (Abcam, #ab33985) 1:1000; anti-β-actin (Sigma #A1978) 1:10,000] and incubated with the membranes for 16 h at 4°C. The HRP-conjugated secondary antibodies [anti-mouse-HRP (Abcam, #ab6709) 1:10,000; anti-rabbit-HRP (Abcam, #ab97051) 1:10,000] were incubated for 60 min at room temperature. The proteins were then visualised using enhanced chemiluminescence (ECL) method (Pierce, #32106) in LAS-3000 unit (Fuji).

### Mitochondrial respiration

Mitochondrial respiration of astrocytes was assessed through Seahorse XFp Analyzer (Agilent) using the Cell MitoStress Test kit (Agilent, #103010-1000). Briefly, astrocytes were seeded in PDL-coated miniplates and experimentally treated before the assay. Phenol red-free Seahorse XF base medium (Agilent, #103335-100) supplemented with 5.5 mm glucose (Sigma, #G8769), 2 mm L-glutamine (ThermoFisher, #G7513), and 1 mm sodium pyruvate (Sigma, #S8636), pH 7.4 was used for the assay. Following manufacturer’s instructions, before each experiment, the Seahorse XFp Analyzer was calibrated, cartridges were hydrated, and treatment compounds were prepared fresh and loaded onto the miniplate ports. Following baseline measurement, primary astrocytes were treated sequentially with 1 μm oligomycin (OM) 4 μm cyanide-4-(trifluoromethoxy) phenylhydrazone (FCCP), and a mixture of antimycin A (AA) and rotenone (Rot; Agilent), 0.5 μm each.

Following each experiment, cells in each well were lysed on ice in RIPA buffer (50 mm Tris-HCl pH 8.0, 150 mm NaCl, 0.1% Triton X-100, 0.5% sodium deoxycholate, 0.1% SDS, 1 mm sodium orthovanadate, and 1 mm NaF) and the protein concentration was determined using BCA assay following the manufacturer’s instructions (ThermoFisher, #23225). Data from each experiment was exported using Wave Software (Agilent) and normalized to blank control wells and to the protein concentration measured for each well. and exported for further statistical analysis.

### 17βHSD10 enzymatic activity assay

The enzymatic activity of 17βHSD10 measured using the fluorogenic probe (−)-cyclohexenyl amino naphthalene alcohol [(−)-CHANA; Muirhead et al., 2010]. The oxidation of this molecule results in the formation of a highly fluorescent ketone cyclohexenyl amino naphthalene ketone (CHANK). Furthermore, the selectivity of the probe has been previously confirmed in HEK293T cells, where showed minimal fluorescence in control and high metabolism in 17βHSD10-overexpressing cells (Muirhead et al., 2010). CHANA was kindly provided by Dr. Laura Aitken (University of St Andrews), reconstituted in DMSO and further diluted into standard astrocyte cell culture medium buffered with 10 mm HEPES before the experimental procedure. Cells were seeded into in 96-well black wall plates, experimentally treated and exposed to CHANA with final assay concentration of 20 μm. The conversion of CHANA to CHANK was measured over 90 min at 37°C using FLUOstar Optima microplate reader (BMG; excitation wavelength 380 nm, emission wavelength 520 nm). The assay quality was ensured for each experiment by, calculating Z’ values:

$$Z^{\prime }=1-\frac{3\sigma_{p}+3\sigma_{n}}{\left|\mu_{p}+\mu_{n}\right|},$$

where the SDs (σ) and the means (μ) of triplicate positive (p) and negative (n) controls were measured. Z’ values above 0.5 are accepted to indicate reliable activity detection and excellent assay quality (Zhang et al., 1999) and only Z’ values between 0.6 and 0.8 were used for the currently reported results. The slope of fluorescence intensity change generated by CHANA to CHANK metabolism was calculated and used as 17βHSD10 activity indicator. All values were normalized and shown as % change from control cells and presented as “CHANA turnover” throughout the Results.

### Mitochondrial morphology measurement and colocalization analysis

The mitochondrial network morphology was assessed using the ImageJ-supported toolset MiNA (Mitochondrial Network Analysis) developed by Valente et al. (2017). The source code for the analysis is kindly provided by the authors in GitHub repository: https://github.com/StuartLab/MiNA/tree/master.

Confocal microscopy images of MitoTracker Red CMXRos-stained mitochondrial networks were preprocessed to obtain high-contrast images permitting for the reliable quantification of mitochondrial network morphology in ImageJ. The output of MiNA was used to evaluate branching morphologies exhibited by mitochondrial networks of primary astrocytes. Optimizing the analysis protocol for this model allowed to distinguish between unbranched structures (puncta and rods) versus branched network formations (Valente et al., 2017). Briefly, the workflow started with the conversion of the images to binary, skeletonizing and analyzing each skeleton. The currently described analysis uses four parameters discussed in the original paper by Valente et al. (2017) to characterize mitochondrial structure: (1) mitochondrial footprint: showing the area of detected mitochondrial signal (μm^2^); (2) mean branch length: indicating the average length of all the lines used to represent the mitochondrial structures (μm); (3) total branch length: showing the mean of the sum of the lengths of branches for each independent structure representing part of the mitochondrial skeleton in a single cell (μm); (4) network branching: showing the mean number of attached lines used to represent each structure.

Colocalization of 17βHSD10 and the astrocytic marker GFAP was quantified using confocal images of acute cortical, hippocampal, and cerebellar brain slices. The images shown in Extended Data Figure 1-1 were analyzed through the JACoP plugin in ImageJ (Bolte and Cordelières, 2006). The Manders’ coefficient was used to quantify the overlap of the GFAP signal with the 17βHSD10 signal to show what portion of the GFAP-positive cells also express 17βHSD10. Manders’ coefficient is based on Pearson’s correlation coefficient with average intensity values being removed from the equation (Manders et al., 1992). The coefficient varies from 0 (no colocalization) to 1 (100% colocalization; Bolte and Cordelières, 2006).

**Figure 1.**
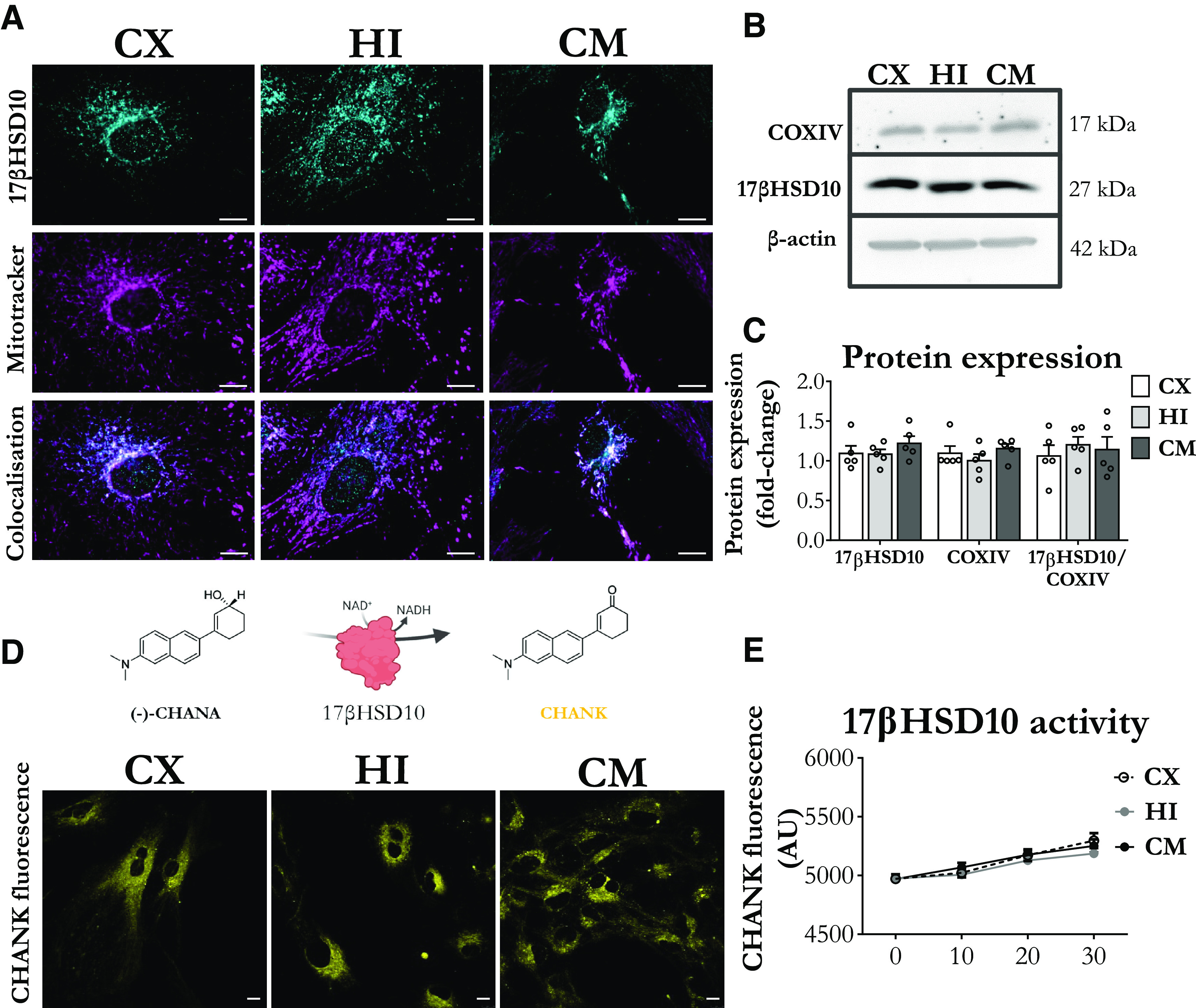
17βHSD10 is expressed and active at a comparable level in astrocytes from the mouse cortex, hippocampus, and cerebellum. ***A***, Representative confocal images of astrocytes immunostained for 17βHSD10 (in cyan) and mitochondrial dye MitoTracker Red CMXRos (in magenta) confirm the mitochondrial localization of the protein. Scale bars: 10 μm. ***B***, Representative immunoblot showing the protein expression levels of 17βHSD10 and mitochondrial COXIV in primary astrocytes with β-actin used as a loading control. ***C***, Quantification of Western blot analysis indicating that both COXIV (*F*_(2,12)_ = 0.93, *p* = 0.42, n.s.) and 17βHSD10 (*F*_(2,12)_ = 0.85, *p* = 0.45, n.s.) maintain comparable levels of expression in astrocytes from the cortex (CX), hippocampus (HI), and cerebellum (CE) and normalizing 17βHSD10 to COXIV also showed no differences between astrocytes (*F*_(2,12)_ = 0.28, *p* = 0.76, n.s.). Bar graphs represent the mean ± SEM; *n* = 5 independent primary cultures; analysis through one-way ANOVA with Tukey’s *post hoc* comparisons. ***D***, Representative confocal images of live astrocytes treated with 20 μm CHANA for 30 min. CHANA is broken down by 17βHSD10 to its fluorescent product CHANK which can be detected by measuring fluorescence levels in the sample (shown in yellow). Scale bar: 10 μm. ***E***, CHANK fluorescence measured over 30 min showed increased fluorescence from baseline in all groups (*F*_(1,45)_ = 88.87, *p* < 0.001) with no differences between astrocytes from the three brain regions (*F*_(2,45)_ = 1.60, *p* = 0.17, n.s; interaction *F*_(2,15)_ = 0.56, *p* = 0.58, n.s.). Analysis through mixed ANOVA with Tukey’s *post hoc* comparisons; n.s, non-significant, *p < 0.05, **p < 0.01, ***p < 0.001. Abbreviations: (-)-cyclohexenyl amino naphthalene alcohol (CHANA), cyclohexenyl amino naphthalene ketone (CHANK), cortex (CX), hippocampus (HI), and cerebellum (CM). Extended Data Figures 1-1, 1-2, 1-3, and 1-4 further show that 17βHSD10 is expressed in uncultured astrocytes of both neonatal and adult mice.

10.1523/ENEURO.0040-22.2022.f1-1 Extended Data Figure 1-1 17βHSD10 is expressed in cortical (CX), hippocampal (HI), and cerebellar (CM) astrocytes of neonatal and adult mice. Low-magnification images of acute brain slices stained for 17βHSD10 (magenta) and astrocytic marker GFAP (cyan) in the frontal cortex, hippocampus, and cerebellar cortex. Colocalization is indicated in white. Colocalization analysis quantified using Manders’ coefficient showing the fraction of GFAP staining overlapping with 17βHSD10 signal is high in cortical (2 d old: M = 0.969; 7 d old: M = 0.901; 2 months old: M = 0.753; 4 months old: M = 0.958), hippocampal (2 d old: M = 0.825; 7 d old: M = 0.711; 2 months old: M = 0.847; 4 months old: M = 0.764), and cerebellar (2 d old: M = 0.954; 7 d old: M = 0.795; 2 months old: M = 0.605; 4 months old: M = 0.648) astrocytes. Scale bar: 300 μm. Download Figure 1-1, EPS file.

### Stress treatments

Oxygen deprivation of primary astrocytes was conducted in a Don Whitley H35 hypoxystation. The oxygen sensors of the incubated station were calibrated before each use and set to 0.5% O_2_, 5% CO_2_ at 37°C according to manufacturer’s instructions. The PBS and culture medium used to wash and treat the cells were preconditioned in the hypoxystation for 1 h before the start of the experiment. The astrocytic cultures were washed with deoxygenated PBS and exposed to 0.5% O_2_ levels, 5% CO_2_ at 37°C for 6 h. The cultures were re-oxygenated by being transferred to normal culture conditions.

Oligomeric Aβ_(1–42)_ was prepared from lyophilized synthetic Aβ_(1–42)_ (1 mg) dissolved in 221.53 μl 1,1,1,3,3,3-hexafluoro-2-propanol (HFIP; Sigma #105228) and dispensed into 10 μl aliquots which were desiccated in a ventilated safety cabinet for 60 min. The resulting peptide films were sealed and stored at −20°C. A final concentration of 5 mm stock was obtained by adding 2 μl DMSO, followed by a 15-min sonication and addition of 48-μl PBS. The sample was vortexed for 30 s and used for treatment of primary astrocytic cultures. The treatment was administered for 48 h before experiments.

### Statistical analysis

Statistical analysis was performed using SPSS Statistics 26 (IBM) while figures were created with GraphPad Prism (GraphPad Software). BioRender was used to create the facilitating figures, including the graphical abstract. The currently presented data constitutes at least three independent biological replicates (i.e., independent primary culture preparations). All data have been tested for normal distribution using Shapiro–Wilk test. Comparisons between more than two groups were conducted using one-way ANOVA, while multiple groups and conditions were compared using two-way or mixed ANOVA. Preceding ANOVA, data were also tested for homoscedasticity using Levene’s test. When conducting repeated measures or mixed ANOVA, Mauchly’s sphericity test was conducted as well. If homoscedasticity or sphericity assumptions were violation, Greenhouse–Geisser corrected values were reported. Since mitochondrial morphology analysis required treating individual astrocytes as datapoints, we utilized a mixed model analysis with culture replicate as a random factor to control for changes that could be attributed to differences between cultures. Tukey’s *post hoc* comparisons were used following ANOVA. The selected confidence interval for statistical significance was 95%; therefore, only *p* < 0.05 was considered significant. Further statistical details are provided in Table 1.

## Results

### Astrocytes from the mouse cortex hippocampus and cerebellum show similar levels of 17βHSD10 expression and activity

Because of the limited amount of literature addressing the role of 17βHSD10 in astrocytes, there had been no evidence of the 17βHSD10 protein being expressed in these cells. While one study suggested that this protein may be expressed in hypertrophic astrocytes surrounding amyloid plaques in AD patients (He et al., 2005), others claimed that 17βHSD10 may not be at all found in murine astrocytes (Fukuzaki et al., 2008). Therefore, our first aim was to confirm whether the protein is present and enzymatically active in astrocytes from different regions of the mouse brain.

We found that 17βHSD10 immunostaining co-localized with mitochondria in cultured mouse astrocytes from the cortex, hippocampus, and cerebellum (Fig. 1*A*). Since previous reports show that total 17βHSD10 protein is higher in the hippocampal as compared with cortical and cerebellar tissue (He et al., 2005), we next asked whether these disparities could be driven by differences in the astrocytic population. The protein expression levels were similar between astrocytes from the three brain regions (Fig. 1*B*,*C*). To confirm that potential differences were not obscured by a variation in mitochondrial levels, COXIV was used as a mitochondrial control. As expected, COXIV was similarly expressed in the three astrocytic samples and normalizing 17βHSD10 levels to COXIV did not reveal further differences (Fig. 1*C*). We provide further immunohistochemical evidence that 17βHSD10 is endogenously expressed in noncultured cortical, hippocampal, and cerebellar astrocytes of neonatal (2 and 7 d of age) and adult mice (two and four months of age; Extended Data Figs. 1-1, 1-2, 1-3, 1-4).

**Figure 2.**
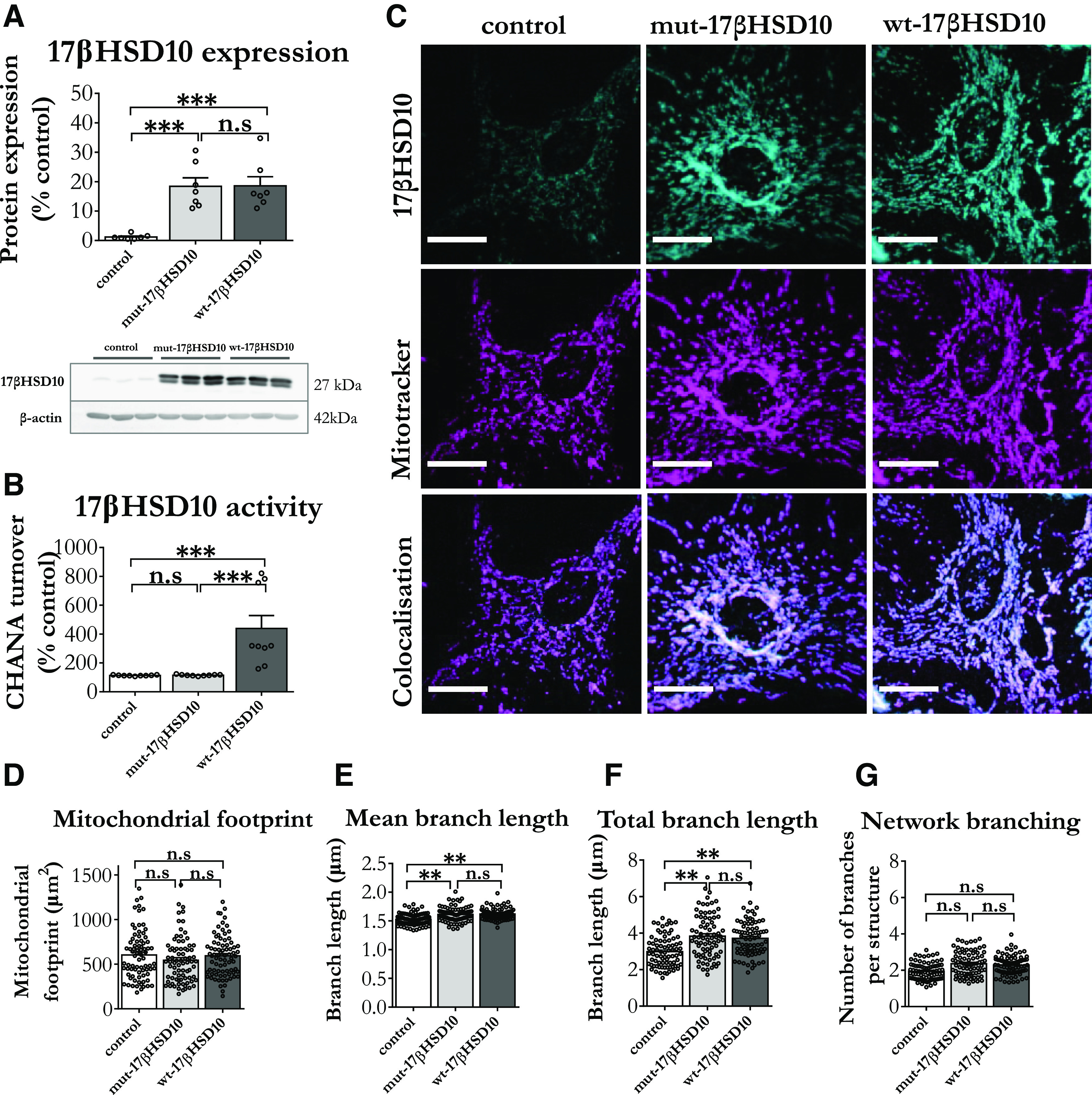
Overexpressing 17βHSD10 in cortical astrocytes induces elongated mitochondrial networks, regardless of 17βHSD10 enzymatic activity. ***A***, 17βHSD10 expression levels were assessed through Western blot analysis 10 d after lentiviral transduction. A representative immunoblot of three independent primary culture preparations and the quantification of 17βHSD10 in these cells showed that the lentiviral protocol induced 20-fold increase in protein expression with both the mutant and wt versions of 17βHSD10 as compared with control astrocytes (*F*_(2,18)_ = 16.67, *p* < 0.001). Bar graphs represent the mean ± SEM. Analysis through one-way ANOVA with Tukey’s *post hoc* comparisons on *n* = 6 independent culture preparations. ***B***, 17βHSD10 enzymatic activity as measured by CHANA turnover rate was significantly increased only when the wt variant of 17βHSD10 was overexpressed, while the mutated version did not cause significant increase (*F*_(2,24)_ = 13.24, *p* < 0.001). Bar graphs represent the mean ± SEM. Analysis through one-way ANOVA with Tukey’s *post hoc* comparisons on *n* = 9 independent culture preparations. ***C***, Representative confocal microscopy images of astrocytes immunostained for 17βHSD10 and mitochondrial dye MitoTracker Red CMXRos confirmed mitochondrial expression of the enzyme in both control and overexpressed conditions. Scale bars: 10 μm. The arrows with dotted lines in the control image indicate predominant mitochondrial morphology in normal conditions; arrows with solid lines in mut-17βHSD10 and wt-17βHSD10 panels indicate abnormal elongated and highly branched network morphology. ***D***, Mitochondrial footprint was uniform across conditions (*F*_(2,751)_ = 0.308, *p* = 0.75, n.s.). ***E***, Mean branch length was greater in mut-17βHSD10 and wt-17βHSD10 populations (*F*_(2,712)_ = 30.16, *p* < 0.004). ***F***, Total branch length increased in the overexpression conditions as compared with control (*F*_(2,715)_ = 212.56, *p* < 0.001) and (***G***) network branching (*F*_(2,715)_ = 1.95, *p* = 0.256) remained unchanged. Bar graphs represent the mean ± SEM. Analysis via mixed model analysis with Tukey’s *post hoc* comparisons; *n* = 3 independent primary culture preparations with 244–254 cells analyzed per condition; n.s, non-significant, ***p < 0.05, **p < 0.01, ***p < 0.001.

10.1523/ENEURO.0040-22.2022.f1-2 Extended Data Figure 1-2 Mouse cortical astrocytes express 17βHSD10. High-magnification images of acute brain slices stained for 17βHSD10 (magenta) and GFAP (cyan). The first column presents the overlay of GFAP and 17βHSD10 staining with colocalization shown in white. 17βHSD10 and GFAP staining alone are observed in the following two columns. The fourth column shows the outlines of the GFAP staining obtained in ImageJ through enhancing (0.25%) and equalizing the contrast of the image followed by the “find edges” function. The final column shows the overlays of the GFAP outlines and 17βHSD10 staining, facilitating the representation of the colocalization. Scale bar: 30 μm. Download Figure 1-2, EPS file.

**Figure 3.**
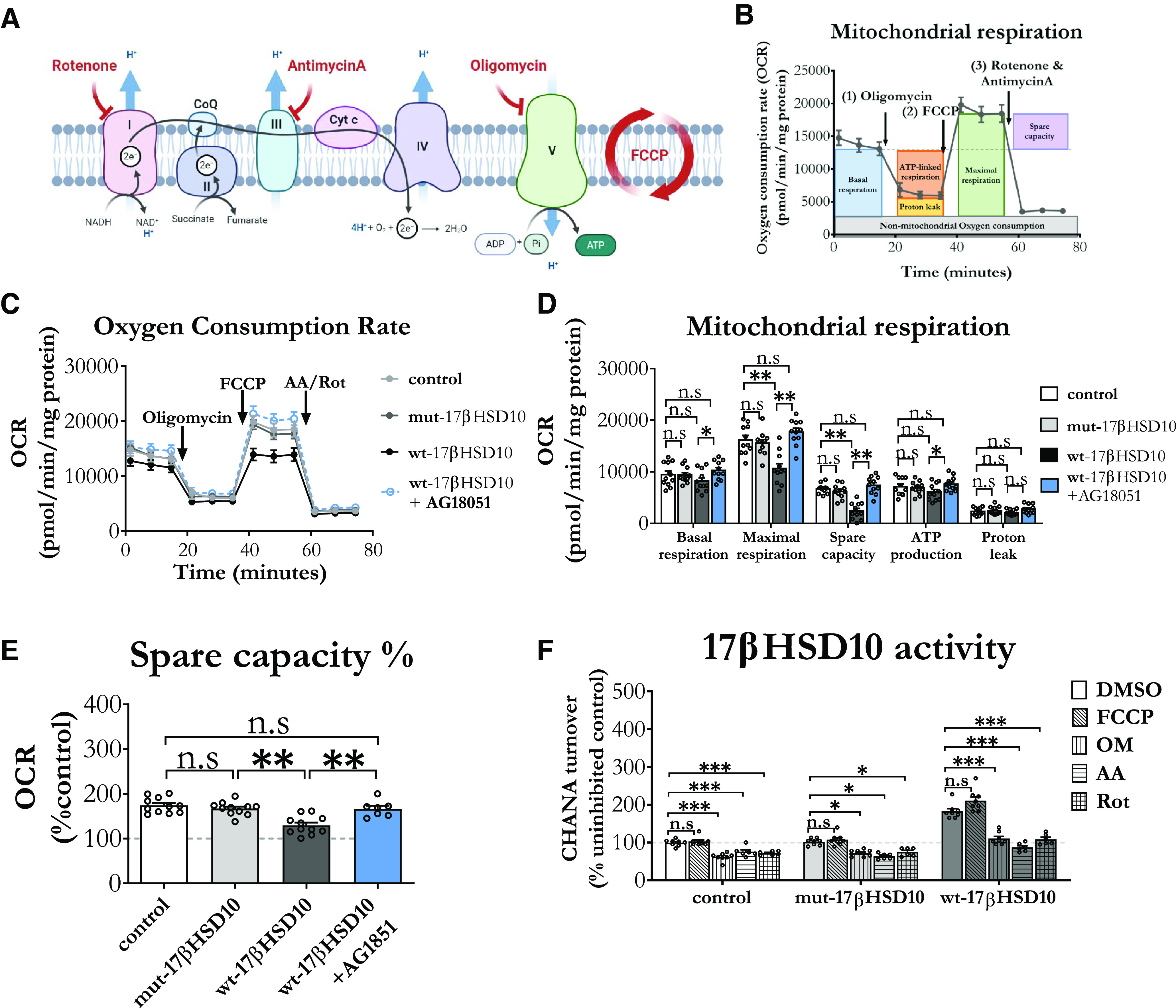
Increased 17βHSD10 activity decreased mitochondrial respiration, while ETC inhibition decreased the activity of the enzyme and AG18051 countered these effects. ***A***, A schematic representation of the ETC targets and utilized inhibitors used for the mitochondrial bioenergetic test. ***B***, Timescale of the experiment, inhibitor administration, and measurement parameters utilized in the respiratory test. ***C***, Mitochondrial respiration was assessed by measuring OCR which was normalized to the protein content in each sample. This profile was further used to calculate the respiratory parameters in the next panel. ***D***, Overexpression of mut-17βHSD10 did not affect the respiratory parameters (*p*s > 0.05); however, wt-17βHSD10 overexpression reduced maximal respiration (*F*_(3,40)_ = 14.22, *p* < 0.001), as well as spare respiratory capacity (*F*_(3,40)_ = 26.737, *p* < 0.001) in cortical astrocytes, while basal respiration (*F*_(3,40)_ = 2.04, *p* = 0.12, n.s.), ATP production (*F*_(3,40)_ = 1.93, *p* = 0.14, n.s.), and proton leak (*F*_(3,40)_ = 1.43, *p* = 0.25, n.s.) remained unaffected. These effects were recovered to baseline when cells were pretreated with 17βHSD10 inhibitor AG18051 (20 μm; *p*s > 0.05). ***E***, Similarly, while all cells with normal activity levels of 17βHSD10 were able to upregulate respiration when challenged, high activity of the protein reduced this metabolic compensation (*F*_(3,40)_ = 13.89, *p* < 0.001). ***F***, Both endogenous and overexpressed 17βHSD10 activity was inhibited by OM, AA, and Rot (control: *F*_(4,30)_ = 29.99, *p* < 0.001; mut-17βHSD10: *F*_(4,25)_ = 13.73, *p* < 0.001 and wt-17βHSD10 (*F*_(4,25)_ = 12.09, *p* < 0.001), while mitochondrial decoupling with FCCP did not affect 17βHSD10 activity (*p*s > 0.05). Graphs represent the mean ± SEM. Compounds: FCCP (carbonyl cyanide-4 (trifluoromethoxy)phenylhydrazone: 4 μm), OM (1.5 μm), AA (5 μm), Rot (5 μm). Treatment time: acute injection. AG18051 (20 μm) was administered for 24 h before the experiment. One-way between-subjects ANOVA with Tukey’s *post hoc* comparisons (*n* = 4–6 independent primary culture preparations); n.s, non-significant, *p < 0.05, **p < 0.01, ***p < 0.001. Abbreviations: cyanide-4-(trifluoromethoxy) phenylhydrazone (FCCP), antimycin A (AA), oligomycin (OM), rotenone (ROT). Extended Data Figure 3-1 shows the effects of AG18051 on respiratory function of astrocytes with endogenous 17βHSD10 levels.

10.1523/ENEURO.0040-22.2022.f3-1 Extended Data Figure 3-1 17βHSD10 inhibition with AG18051 caused upregulated maximal respiration, space respiratory capacity, and proton leak. ***A***, OCR was normalized to the protein content in each sample. This profile was further used to calculate the respiratory parameters in the next panel. ***B***, Although basal respiration and overall ATP production remained unaffected by AG18051 (*t*_(15)_ = 0.99, *p* = 0.338 and *t*_(15)_ = 0.33, *p* = 0.741, respectively), maximal respiration (*t*_(15)_ = 2.30, *p* = 0.036), and spare respiratory capacity (*t*_(15)_ = 3.27, *p* = 0.005) was increased in astrocytes treated with the inhibitor. Treatment time: acute injection. AG18051 (20 μm) was administered for 24 h prior to the experiment. Independent samples *t* tests (*n* = 4–6 independent primary culture preparations); *p < 0.05, **p < 0.01, ***p < 0.001. Download Figure 3-1, EPS file.

Next, we employed an enzymatic assay, which can report 17βHSD10-mediated break-down of CHANA to its fluorescent metabolite CHANK (Muirhead et al., 2010) to compare 17βHSD10 activity between astrocytes (Fig. 1*D*; Materials and Methods, Mitochondrial respiration). No differences were found in the levels of 17βHSD10 activity in astrocytes from the cortex, hippocampus, and cerebellum (Fig. 1*D*,*E*).

Therefore, 17βHSD10 is expressed and enzymatically active in astrocytes from different regions of the murine brain. Any reported 17βHSD10 expression and activity differences between brain regions in normal conditions could not be attributed to astrocytes alone when controlling for total cell numbers and total protein content.

### 17βHSD10 affects mitochondrial network architecture and function in cortical astrocytes

Another important lack in the literature was that while the effects of 17βHSD10 upregulation have been extensively studied in neuronal populations (Lustbader et al., 2004; Tieu et al., 2004; Takuma et al., 2005; Yan and Stern, 2005), there was no information on how the protein impacts astrocytic mitochondria. Therefore, we employed a lentiviral overexpression system to overexpress wt or catalytically inactive 17βHSD10 (mut) into the mitochondria of astrocytes by utilizing a MTS. The mutated variant of the protein harbors a mutation in the catalytic triad (S155, Y168, K172) of the enzyme, replacing Y168 with glycine (Yan et al., 1999; Kissinger et al., 2004), which renders the 17βHSD10 mutant catalytically inactive (mut-17βHSD10; Guest, 2016).

The overexpression of the enzyme was comparable between both wt-17βHSD10 and mut-17βHSD10 (Fig. 2*A*). Overexpression of either variant caused no changes in the viability and mitochondrial superoxide levels, while only wt-17βHSD10 overexpression increased the reported levels of 17βHSD10 activity in cortical astrocytes (Fig. 2*B*).

While confirming the successful mitochondrial targeting of the overexpressed protein (Fig. 2*C*), we also found that overexpression of 17βHSD10, both wt and mut, induced apparent changes in mitochondrial network architecture. In order to test and quantify these observations, we used the ImageJ-supported toolset MiNA (Valente et al., 2017). 17βHSD10 overexpression did not alter the total area occupied by mitochondria (mean area across conditions: 691.21 ± 10.31 μm; Fig. 2*D*), which is consistent with the equal mitochondrial number suggested by the mitochondrial loading control (COXIV) reported in Figure 1. On the other hand, branch parameters indicated that mitochondrial networks with overexpressed wt-17βHSD10 and mut- 17βHSD10 have increased length of individual structures by 16% (Fig. 2*E*) and elongation of branched mitochondrial structures by >40% (Fig. 2*F*). However, the number of mitochondrial network branches remained unchanged (Fig. 2*G*). These findings were consistent with elongated mitochondrial phenotype, pointing to altered mitochondrial dynamics.

### Overexpression of catalytically active, but not mutated/inactive, 17βHSD10 inhibits mitochondrial respiration in cortical astrocytes

After characterizing our model systems, we proceeded to addressing the effect of wt-17βHSD10 and mut-17βHSD10 overexpression on mitochondrial respiration in cortical astrocytes (Fig. 3). Utilizing mitochondrial stress testing (Fig. 3*A*), we aimed to assess the effects of 17βHSD10 overexpression on basal mitochondrial respiration and the capability of astrocytes to respond to an energetic demand by upregulating mitochondrial respiration.

The first finding was that the overexpression of overexpressed wt-17βHSD10 did not affect mitochondrial respiration (Fig. 3*C*,*D*). However, it decreased maximal respiratory capacity by 45% from control (decrease by 5539 ± 931 pmol/min/mg protein) and spare respiratory capacity by 65% (decrease by 4306 ± 610 pmol/min/mg protein), without affecting basal respiratory capacity, ATP production and proton leak (Fig. 3*D*). Control and mut-17βHSD10-overexpressing cells were able to upregulate their respiration by almost 70% from baseline. While wt-17βHSD10-overexpressing cells were also able to increase their respiration when challenged to reach maximum oxygen consumption rate (OCR) through FFCP administration which induced maximal oxygen consumption at Complex IV (*t*_(20)_ = 2.145, *p* = 0.044), this increase was significantly reduced as compared with the other two conditions, reaching only 30% above baseline (Fig. 3*E*).

In order to confirm whether all the respiratory changes associated with wt-17βHSD10 overexpression were caused by the excess enzymatic activity, we employed a known 17βHSD10 inhibitor. AG18051 which is currently the most potent available 17βHSD10 inhibitor (IC_50_ 92 nm; Kissinger et al., 2004; Vinklarova et al., 2020). When primary astrocytes overexpressing wt-17βHSD10 were pretreated with 20 μm AG18051, all respiratory parameters were restored to control levels (Fig. 3*C–E*). This further confirmed that the excess enzymatic activity of the protein was the main driver of respiratory inhibition in these cells. Furthermore, the inhibitor increased maximal respiration and spare respiratory capacity in cortical astrocytes with endogenous levels of 17βHSD10 (Extended Data Fig. 3-1).

We next sought out to find whether mitochondrial decoupling or respiratory inhibition through different ETC enzymes would affect 17βHSD10 activity. Mitochondrial decoupling though addition of FCCP did not affect 17βHSD10 activity (Fig. 3*F*). However, inhibition of respiration through Complex I (via Rot), Complex III (via AA), and Complex V (using OM), caused a significant decrease in 17βHSD10 activity in all astrocytes (Fig. 3*F*; Extended Data Fig. 3-1).

Therefore, our findings suggest that excessive 17βHSD10 activity decreases maximal astrocytic respiration and spare respiratory capacity, without affecting baseline respiratory levels. However, inhibition of ETC Complex I, III, or V causes activity down-regulation of both endogenous and overexpressed 17βHSD10 (both mut and wt). Altogether, these findings suggest that while 17βHSD10 activity is dependent on ETC activity, overexpressed activity of the enzyme could inhibit the ability of these cells to upregulate their mitochondrial respiration to meet a sudden increase in energy demand.

### Ischemia-reoxygenation increases 17βHSD10 activity and expression in astrocytes, affecting ROS generation and mitochondrial network morphology

Ischemic conditions during stroke are characterized by the disruption of oxygen and nutrient supply to the affected part of the brain. The complex nature of this insult implicates varying degrees of neurodegeneration, and the recovery is heavily dependent on astrocyte mediated-nutrient support to neurons (Rossi et al., 2007; Chaitanya et al., 2014). One early study by Yan et al. (2000) found that 17βHSD10 expression is elevated in neurons proximal to the infracted area in a mouse stroke model. Overexpressing 17βHSD10 in this model was found to enhance TCA flux and ATP levels, while decreasing neurologic deficit and infarction volume (Yan et al., 2000). The relevance of astrocytes, which are a major factor in the recovery from this type of injury was never addressed (Rossi et al., 2007; Guo et al., 2012; Chaitanya et al., 2014).

**Figure 4.**
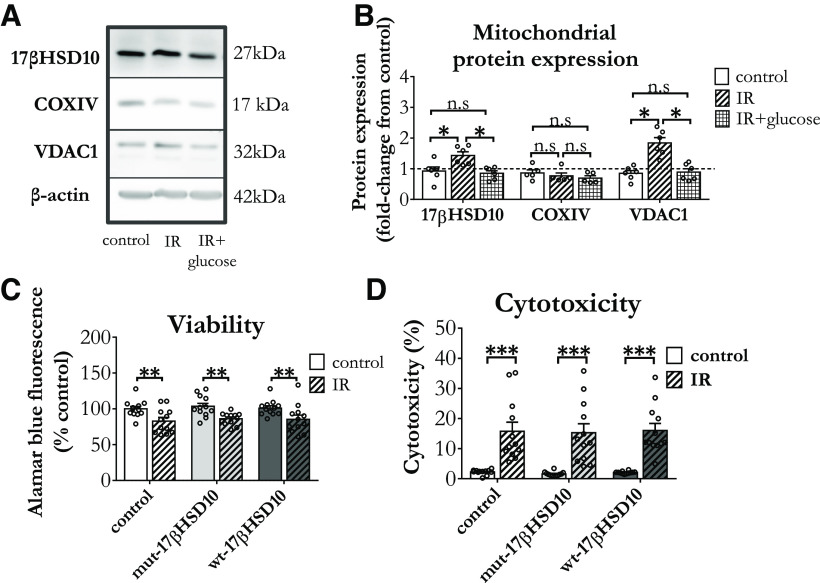
17βHSD10 expression and activity increased following ischemia-reoxygenation (IR) insult, affecting superoxide generation and mitochondrial network morphology. ***A***, Western blot analysis of mitochondrial protein expression showed (***B***) that 17βHSD10 (*F*_(2,15)_ = 7.61, *p* = 0.005) was increased following IR in the absence of glucose, with VDAC1 following similar pattern (*F*_(2,15)_ = 18.94, *p* < 0.001), while COXIV remained stable (*F*_(2,12)_ = 0.88, *p* = 0.44, n.s.). ***C***, IR reduced viability (treatment main effect: *F*_(1,66)_ = 72.12, *p* < 0.001) to a similar level in all astrocytes (17βHSD10 expression main effect: *F*_(2,66)_ = 0.06, *p* = 0.94, n.s.). ***D***, Cytotoxicity was increased by the insult (treatment main effect: *F*_(1,66)_ = 24.15, *p* < 0.001) with all three groups showing similar change (17βHSD10 expression main effect: *F*_(2,66)_ = 0.32, *p* = 0.73, n.s.). Analysis via two-way between subjects ANOVA with Tukey’s *post hoc* test, *n* = 4 independent biological replicates; n.s, non-significant, *p < 0.05, **p < 0.01, ***p < 0.001. Abbreviations: ischemia-reoxygenation (IR), Voltage Dependent Anion Channel 1 (VDAC1), Cytochrome c oxidase (COX). Treatment: hypoxia, 6 h (0 mm glucose 0.5% O_2_; 5% CO_2_ at 37°C) followed by 2 h of reoxygenation.

Therefore, we employed a simplified *in vitro* stroke model to address this lack in literature. Primary cortical astrocytes were incubated in a hypoxystation with 0.5% O_2_, 5% CO_2_ at 37°C for 6 h and returned to normal conditions for 2 h before assessment. When glucose (5.5 mm) was present in their environment, 17βHSD10 levels remained similar to control (Fig. 4*A*,*B*). However, 17βHSD10 protein levels were elevated when no glucose was present during IR. Although COXIV expression levels remained stable in these conditions, VDAC1 was increased in the absence of glucose. These findings are consistent with the knowledge that the mitochondrial permeability transition pore (mPTP) opens following IR injury (Reichert et al., 2001) and while the participation of VDAC1 in mPTP formation following IR is controversial (McCommis and Baines, 2012), VDAC1 upregulation is known to promote apoptotic activation following IR (Stary et al., 2016; Yang et al., 2021). The presence of glucose in these conditions alleviates the energetic crisis in the model, ensuring that astrocytes can rely on anaerobic glycolysis for their survival (Dienel and Hertz, 2005; Hertz, 2008). This metabolic supplementation prevented the increase of both 17βHSD10 and VDAC1 expression levels (Fig. 4*A*,*B*).

In addition to altering mitochondrial protein expression, IR caused a decrease in astrocytic viability (Fig. 4*C*) with a concomitant increase in cytotoxicity as measured by LDH release (Fig. 4*D*), while overexpressing 17βHSD10 did not affect these changes. Although the timescale of the current experiment did not assess long-lasting effects on astrocytes, the current observations suggest that 17βHSD10 might be a crucial factor for metabolic recovery following such an injury.

**Figure 5.**
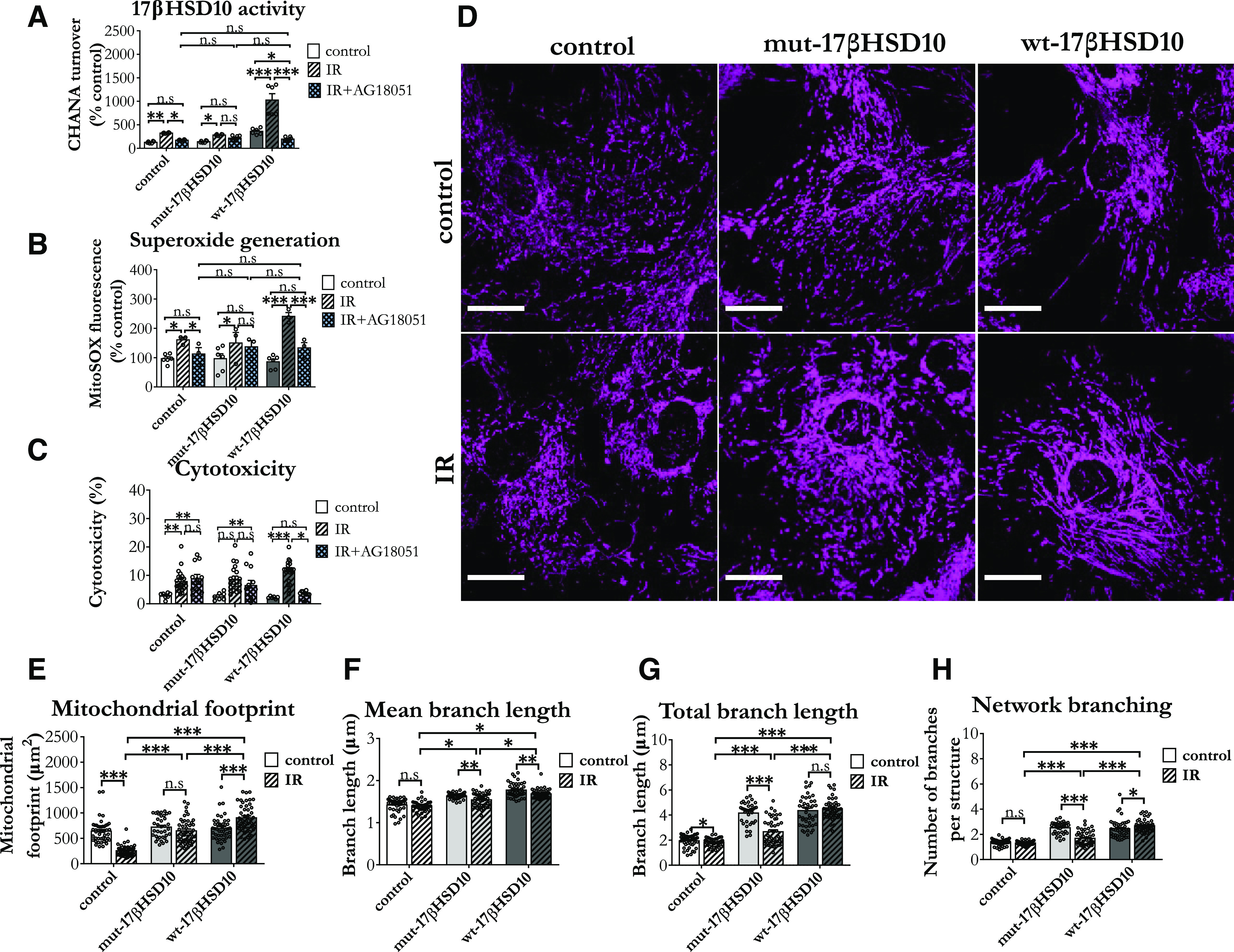
17βHSD10 activity increased following IR and this affected superoxide generation and mitochondrial network morphology. ***A***, 17βHSD10 activity increased following IR in all astrocytes and AG18051 inhibited this elevation (*F*_(4,45)_ = 20.88, *p* < 0.001); *n* = 3 independent primary culture preparations. Analysis via two-way between subjects ANOVA with Tukey’s *post hoc* test. ***B***, Superoxide generation was increased following the insult and the effects were ameliorated by AG18051 in all astrocytes but mut-17βHSD10-overexpressing cells. The increase was of higher magnitude in wt-17βHSD10-overexpressing cells, and the AG18051-mediated decrease was particularly pronounced in this condition (interaction: *F*_(4,27)_ = 3.18, *p* = 0.029). Analysis on *n* = 3 independent biological replicates using two-way between subjects ANOVA with Tukey’s *post hoc* test. ***C***, AG18051 did not rescued the cytotoxic effect induced by IR only in astrocytes overexpressing wt-17βHSD10 (interaction: *F*_(4,111)_ = 6.03, *p* < 0.001). ***D***, Representative confocal images of mitochondrial networks in astrocytes in control and IR conditions. Arrows with dotted line indicate fragmented mitochondrial morphology with reduced branching, while arrows with solid line show elongated and highly branched network morphology. Scale bars: 10 μm. ***E***, Mitochondrial footprint changes following IR depended on 17βHSD10 expression and catalytic activity (interaction between treatment and 17βHSD10 expression phenotype: *F*_(2,243)_ = 96.80, *p* < 0.001) with substantial reduction in normal astrocytes, nonsignificant effect in mut-17βHSD10-overexpressing cells and an increase in the wt-17βHSD10-overexpressing group. ***F***, Average branch length decreased following IR and this was significant in 17βHSD10-overexpressing astrocytes (main effect of 17βHSD10 expression: *F*_(2,243)_ = 14.29, *p* = 0.015). ***G***, Total branch length changes following IR depended on 17βHSD10 expression and catalytic activity (interaction between treatment and 17βHSD10 expression phenotype: *F*_(2,261)_ = 7.77, *p* = 0.041) with only wt-17βHSD10-overexpressing astrocytes resisting the decrease in this parameter following IR insult. ***H***, Network branching also showed significant interaction between treatment and 17βHSD10 expression phenotype (*F*_(2,243)_ = 22.14, *p* = 0.007), whereby wt-17βHSD10 showed an increase of branch number following treatment. Analysis via two-way between subjects ANOVA with Tukey’s *post hoc* test, *n* = 3 independent biological replicates, 45 cells per condition; n.s, non-significant, *p < 0.05, **p < 0.01, ***p < 0.001. Abbreviations: ischemia-reoxygenation (IR). Treatment: hypoxia, 6 h (0 mm glucose 0.5% O_2_; 5% CO_2_ at 37°C) followed by 2 h of reoxygenation.

As expected, based on observed protein expression shifts, the detected 17βHSD10 activity increased following an IR insult, and this change was evident in normal, as well as astrocytes with overexpressed mut-17βHSD10 and wt-17βHSD10 (Fig. 5*A*). These changes were mirrored by mitochondrial superoxide generation, whereby IR induced an increase in all cells, but the effect was particularly pronounced in those with overexpressed wt-17βHSD10 (Fig. 5*B*). Importantly, both enzymatic activity elevation and superoxide generation were countered by the addition of AG18051. However, the inhibitor reduced IR-induced cytotoxicity only in wt-17βHSD10-overexpressing astrocytes (Fig. 5*C*).

The current results were also consistent with the literature showing that cerebral ischemia causes increased mitochondrial fission and increased mitophagy in astrocytes following IR (Lan et al., 2018; Quintana et al., 2019). The phenotype we observed in normal astrocytes exposed to IR is consistent with mild mitochondrial fragmentation (Fig. 5*F–H*) and pronounced reduction of mitochondrial footprint (Fig. 5*E*). Interestingly, although overexpression of either wt-17βHSD10 or mut-17βHSD10 caused mitochondrial network elongation with a hyperfused phenotype, mut-17βHSD10 overexpression did not prevent mitochondrial fragmentation in astrocytes exposed to IR, while the opposite was seen with wt-17βHSD10 overexpression. Additionally, mut-17βHSD10 overexpression inhibited the decrease of mitochondrial territory observed in normal astrocytes exposed to IR. On the other hand, wt-17βHSD10 overexpression not only halted the decrease, but further increased mitochondrial footprint following the insult (Fig. 5*E*). What is more, wt-17βHSD10-overexpressing cells did not exhibit the characteristic mitochondrial fragmentation (Fig. 5*F–H*).

Collectively, our data suggested that mut-17βHSD10 overexpression caused a mitochondrial hyperfused phenotype at baseline, and was not able to counter fragmentation associated with IR. The wt-17βHSD10 overproduction not only countered mitochondrial fragmentation but potentiated mitochondrial elongation following IR. Excessive 17βHSD10 activity also increased mitochondrial superoxide generation in IR conditions.

There is a debate about whether IR-induced mitochondrial reduction is beneficial or not. However, the consensus maintains that it would depend on the severity of the injury (Shao et al., 2020). Although the current results do not address the underlying mechanisms, we report that abnormally high 17βHSD10 enzymatic activity (following wt-17βHSD10 overexpression) potentiates ROS, alters network morphology, and halts mitochondrial reduction in cortical astrocytes exposed to IR. We speculate that the phenotype is likely to affect the mitochondrial network balance and hamper the recovery following this type of injury; however, further mechanistic studies should address these phenomena in more detail.

### Oligomeric Aβ_(1–42)_ alters 17βHSD10 expression and activity in astrocytes, inducing ROS generation and alterations in mitochondrial network architecture

Until now, the literature studying the role of 17βHSD10 in AD has mainly focused on neuronal cells, where Aβ binds 17βHSD10 inducing a number of neurotoxic effects (Yan et al., 1999; Lustbader et al., 2004; Takuma et al., 2005; Yan and Stern, 2005; Borger et al., 2011). However, non-neuronal populations such as astrocytes provide essential metabolic support to the brain and clear amyloid plaques from the brain. While soluble Aβ_(1-42)_ oligomers rather than fibrils have been proposed to drive the main toxic events in AD pathology, 17βHSD10 also has a higher affinity for binding Aβ_(1-42)_ as compared with Aβ_(1–40)_ (Chen et al., 2017; De et al., 2019; Ciudad et al., 2020) and the affinity is determined by the oligomerization of the peptide (Hemmerová et al., 2019). In line with these studies, we opted for utilizing oligomeric Aβ_(1-42)_ treatment in astrocytes.

**Figure 6.**
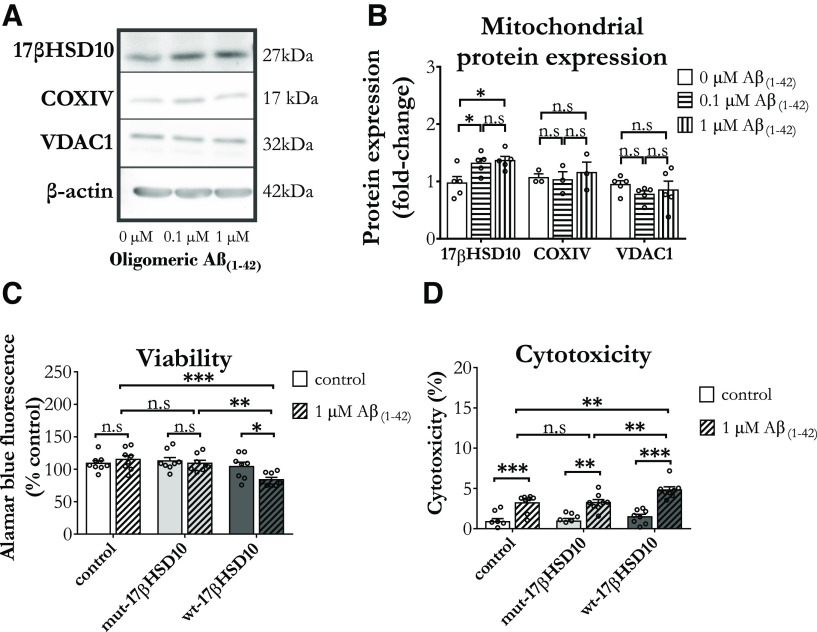
Oligomeric Aβ_(1-42)_ increased 17βHSD10 expression in cortical astrocytes, while overexpression of wt-17βHSD10 exacerbated Aβ_(1-42)_-induced cell death. ***A***, Representative immunoblot showing alterations in mitochondrial protein expression following a 48-h treatment with 0–1 μm oligomeric Aβ_(1-42)_. ***B***, Quantitative analysis showed that oligomeric Aβ_(1-42)_ elevated 17βHSD10 expression (*F*_(2,12)_ = 5.65, *p* = 0.019), while the other two proteins were not affected (COXIV: *F*_(2,12)_ = 0.22, *p* = 0.81, n.s.; VDAC1: *F*_(2,12)_ = 0.67, *p* = 0.53, n.s.). Analysis via one-way between subjects ANOVA with Tukey’s *post hoc* test on *n* = 3 independent primary culture preparations. ***C***, Viability was significantly reduced only in wt-17βHSD10-overexpressing astrocytes treated with Aβ_(1-42)_ (interaction between 17βHSD10 expression and treatment *F*_(2,48)_ = 3.36, *p* = 0.044). ***D***, Cytotoxicity was elevated by Aβ_(1-42)_ in all astrocytes (main effect of treatment: *F*_(1,48)_ = 80.08, *p* < 0.001) and wt-17βHSD10 expression particularly potentiated this increase (main effect of 17βHSD10 expression: *F*_(2,48)_ = 5.95, *p* = 0.005); two-way between subjects ANOVA on *n* = 4 biological replicates. Graphs shows mean ± SEM; n.s, non-significant, *p < 0.05, **p < 0.01, ***p < 0.001. Abbreviations: ischemia-reoxygenation (IR), Voltage Dependent Anion Channel 1 (VDAC1), Cytochrome c oxidase (COX).

First, we confirmed that similar to observations in neurons and total expression in cortical matter (Yan et al., 2000; Lustbader et al., 2004; Yan and Stern, 2005), cortical astrocytes exposed to oligomeric Aβ_(1-42)_ upregulated their 17βHSD10 protein levels, without affecting COXIV and VDAC1 (Fig. 6*A*,*B*). Importantly, the overexpression of wt-17βHSD10 also caused a significant decrease of viability in an amyloid-rich environment (Fig. 6*C*). Cytotoxicity levels were significant in all cells treated with amyloid; however, the overall magnitude did not exceed 5% (Fig. 6*D*).

Yan et al. (1999) reported that the currently studied mutated variant of 17βHSD10 (Y168G), can bind Aβ_(1-42)_ with similar affinity to the wt variant. This allowed us to study the impact of 17βHSD10 overexpression in astrocytes in an amyloid-rich environment, while only eliminating the activity-mediated aspect of the interaction. Interestingly, we found that cortical astrocytes overexpressing the catalytically inactive form of the protein (mut-17βHSD10) showed similar response to amyloid in the context of viability, 17βHSD10 activity, ROS generation, mitochondrial territory and morphology.

**Figure 7.**
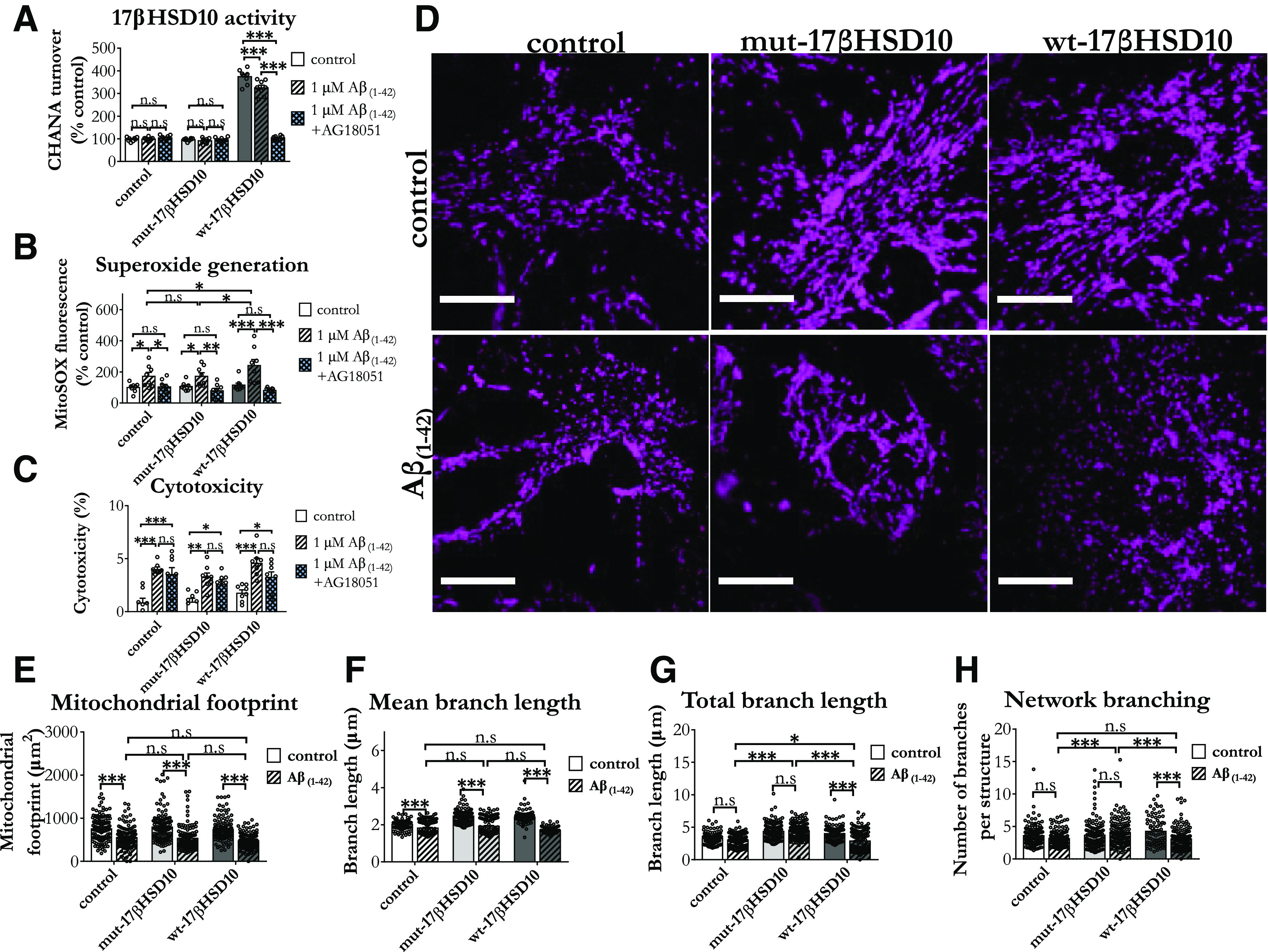
The catalytic activity of overexpressed 17βHSD10 decreased following treatment with oligomeric Aβ_(1-42)_, and this was associated with exacerbated superoxide generation and mitochondrial fragmentation. ***A***, Oligomeric Aβ_(1-42)_ reduced detected 17βHSD10 activity only in wt-17βHSD10-overexpressing astrocytes, and AG18051 co-administration abolished this difference (interaction between treatment and 17βHSD10 expression: *F*_(4,63)_ = 178.45, *p* < 0.001). ***B***, Oligomeric Aβ_(1-42)_ increased superoxide generation in all astrocytes and the effect was of greatest magnitude in the wt-17βHSD10-overexpressing group, while AG18051 countered the increase in all conditions (*F*_(2,63)_ = 10.34, *p* < 0.001). Bar graphs show mean ± SEM; *n* = 3 independent primary cultures; two-way between subjects ANOVA with Tukey’s *post hoc* comparisons. ***C***, AG18051 did not rescue the cytotoxic effect induced by Aβ_(1-42)_ (*p*s > 0.05). ***D***, Representative confocal images of astrocytic mitochondrial networks in control and Aβ_(1-42)_ treatment. Arrows with dotted line indicate fragmented mitochondrial morphology which was particularly pronounced in wt-17βHSD10-overexpressing astrocytes. Scale bars: 10 μm. ***E***, Mitochondrial footprint was reduced in all astrocytes following amyloid treatment (treatment main effect: *F*_(1,702)_ = 30.79, *p* = 0.031) in all three groups of astrocytes (*F*_(2,702)_ = 0.50, *p* = 0.640). ***F***, Changes in mean branch length depended on both 17βHSD10 expression and amyloid treatment (interaction *F*_(2,702)_ = 21.17, *p* = 0.007). ***G***, Total branch length also displayed a significant interaction (*F*_(2,702)_ = 12.58, *p* = 0.019) whereby amyloid reduced summed branch length only in cells overexpressing the catalytically active form of the protein. ***H***, Network branching also showed significant interaction between treatment and protein expression (*F*_(2,702)_ = 13.59, *p* = 0.016) with only wt-17βHSD10-overexpressing cells showing significant decrease in the number of mitochondrial branches following amyloid treatment. Analysis via two-way between subjects ANOVA with Tukey’s *post hoc* test, *n* = 3 biological replicates with 120 cells analyzed per treatment; n.s, non-significant, *p < 0.05, **p < 0.01, ***p < 0.001. Treatments: Aβ_(1-42)_ 1 μm for 48 h; AG18051 20 μm.

Consistent with previous reports in neurons showing that Aβ_(1-42)_ inhibits 17βHSD10 activity, while increasing its expression levels (Yan et al., 1999; Yan and Stern, 2005), we found that in cortical astrocytes amyloid rich environment induces elevated 17βHSD10 expression. However, when wt-17βHSD10 was overexpressed, Aβ_(1-42)_ was able to reduce the activity of the enzyme as compared with vehicle control (Fig. 7*A*), while no such differences were observed in cells with endogenous levels of 17βHSD10 activity. Furthermore, inhibition of 17βHSD10 activity via AG18051 (Fig. 7*A*) inhibited the elevated superoxide generation caused by amyloid in all astrocytes (Fig. 7*B*). Cortical astrocytes overexpressing the catalytically active form of the enzyme also showed exacerbated elevation in superoxide postamyloid treatment; however, this was also alleviated by AG18051 treatment. However, AG18051 did not reduce amyloid-induced cytotoxicity (Fig. 7*C*), regardless of 17βHSD10 expression and activity.

We further studied mitochondrial architecture in these cells and found that Aβ_(1-42)_ reduced mitochondrial territory in all astrocytes (Fig. 7*E*). While mitochondrial fragmentation was also observed in all amyloid-treated samples, the phenotype was particularly exacerbated when wt-17βHSD10 was overexpressed in cortical astrocytes (Fig. 7*F–H*).

## Discussion

Astrocytes play an essential role in neuroprotection and recovery from ischemic insult (Guo et al., 2012; Zamanian et al., 2012; Cai et al., 2020) and AD-associated metabolic and amyloidogenic stress (De Strooper and Karran, 2016; Liddelow et al., 2017; Grubman et al., 2019; Mathys et al., 2019). Although their energy metabolism has multiple implications in these pathologies, the role of their mitochondria has been generally overlooked since the majority of their ATP is derived through glycolysis (Pellerin and Magistretti, 1994; Magistretti and Pellerin, 1999; Magistretti, 2006). It is now becoming clear that mitochondria are crucial for astrocytic metabolic flexibility during metabolic challenges, malignant transitions, and region-dependent vulnerabilities to neurodegeneration (Polyzos et al., 2019; Ryu et al., 2019; Dematteis et al., 2020; Habib et al., 2020). The current study provides novel insights into the role of astrocytic mitochondria in pathology by showing that 17βHSD10, a hypothesized metabolic switch between energy and neurosteroid metabolism (Borger et al., 2011), has increased expression and catalytic activity following metabolic and amyloidogenic stress, which subsequently affects their mitochondrial function.

Existing reports on the role of 17βHSD10 in astrocytes are predominantly theoretical, however they propose that the protein is upregulated in astrocytes surrounding amyloidogenic plaques, which might disrupt steroid metabolism in the brains of AD patients (He et al., 2005; He and Yang, 2006). Our findings support the idea that 17βHSD10 has an important role in astrocytic metabolism and neurodegenerative pathology. We further found that astrocytes from the murine cortex, hippocampus, and cerebellum express enzymatically active 17βHSD10 at comparable levels. Furthermore, overexpressing either the normal or the mutated (catalytically inactive) version of the enzyme did not alter the viability (Fig. 2*A*), baseline mitochondrial respiration (Fig. 3*D*) and ROS generation (Figs. 5*B*, 7*B*) in cortical astrocytes under normal conditions. However, mitochondrially targeted overexpression of the protein, regardless of its catalytic activity, induced elongated mitochondrial architecture without affecting overall mitochondrial content these cells. Indeed, such elongation has been documented by Bertolin et al. (2015), who showed that 17βHSD10 promotes this phenotype by interfering with Drp1 recruitment to mitochondria. We further extended these findings by showing that the mechanism is not dependent on the catalytic activity of 17βHSD10, and that the phenomenon also impacts mitochondrial territory and network architecture during stress. However, the exact mechanistic underpinnings of these observations must be addressed in future studies.

Inhibition of mitochondrial ETC Complexes I, III, and V decreased 17βHSD10 enzymatic activity. However, overexpression of wt-17βHSD10 caused a decline in the ability of astrocytes to upregulate their respiration when metabolically challenged. Unlike the overexpression of the wt variant, the catalytically inactive form did not affect maximal respiration and spare respiratory capacity. The hypothesis that respiratory inhibition is catalytically-dependent was confirmed by the fact that AG18051, a potent 17βHSD10 inhibitor (Kissinger et al., 2004), countered respiratory inhibition in cortical astrocytes overexpressing wt-17βHSD10 and caused a small increase in spare respiratory capacity in astrocytes with endogenous 17βHSD10 levels. These findings are not consistent with existing literature on cancer biology showing that 17βHSD10 overexpression in PC12 cells increases their mitochondrial respiration through Complex IV activity upregulation (Carlson et al., 2015).

However, other studies found that mitochondrial respiration in neurons of 17βHSD10-overexpressing mice remains similar to control (Tieu et al., 2004; Takuma et al., 2005), while amyloid-rich environment induces a Complex IV-mediated inhibition of the process (Takuma et al., 2005). In contrast, 17βHSD10 overexpression increases respiration and ATP production in mouse models of Parkinson’s disease (Tieu et al., 2004) and ischemia (Yan et al., 2000). Therefore, the general discrepancy in the literature is related to the cell type and the metabolic demands under which 17βHSD10 is being studied. Furthermore, most of the literature characterizing 17βHSD10 impact on respiration utilized methodology where respiratory complex activity was measured in lysed samples through spectrophotometric methodology (Tieu et al., 2003; Takuma et al., 2005; Carlson et al., 2015), rather than intact cells as was performed in the current study. Importantly, ETC configuration in astrocytes is reportedly characterized by higher levels of free Complex I, while in neurons Complex I and Complex III are assembled into supercomplex formations. Previously published evidence suggests that these differences render the astrocytic ETC less “efficient” and produces higher levels of ROS in respiration (Lopez-Fabuel et al., 2016). Such differences could explain why the current observations are not consistent with data in cancer or neuronal cell models, further emphasizing that the role of 17βHSD10 could be dependent on cell type and specific metabolic conditions and stresses.

While 17βHSD10 enzymatic activity was dependent on ETC function in cortical astrocytes, excessive activity of the enzyme impaired the ability of these cells to upregulate their respiration during metabolic challenge. These changes could have profound impact on astrocytic function and survival in stress conditions associated with disease. As an example of such a malignant state, ischemic insult causes severe bioenergetic stress which forces astrocytes to use their glycogen reserves (Cai et al., 2020; Köhler et al., 2021), while reoxygenation imposes a severe load on mitochondrial respiratory chain, driving reverse electron transport through Complex II, causing elevated ROS generation in the form of superoxide generation at Complex I (Chouchani et al., 2014). Interestingly, our results showed that ischemia elevated 17βHSD10 expression and activity, however hypoxia in the presence of glucose did not. These observations were expected, since astrocytes are capable of anaerobic glycolysis to ensure lactate production for energy requirements and neuronal supply (Dienel and Hertz, 2005; Hertz, 2008). Therefore, 17βHSD10 might be particularly relevant in conditions where astrocytes cannot rely on glycolytic metabolism for their survival, suggesting that the enzyme is particularly important for metabolic flexibility in conditions of reduced glucose supply regardless of aerobic or anaerobic conditions.

Consistent with literature, IR-exposed astrocytes had increased superoxide generation (Kowaltowski and Vercesi, 1999), increased voltage-dependent anion channel 1 (VDAC1) expression (Yang et al., 2021) and mitochondrial network fragmentation while decreasing mitochondrial territory (Lan et al., 2018; Quintana et al., 2019). The elimination of damaged mitochondria following ischemic stroke is crucial for recovery (Lan et al., 2018) and it is activated through several mechanisms, which include ischemia-induced mPTP opening, mitochondrial membrane potential disruption (Reichert et al., 2001; Wang et al., 2012), ROS elevation (Wang et al., 2012; Fan et al., 2019), and Drp1-mediated activation (Zuo et al., 2014).

However, the overexpression of catalytically active 17βHSD10 (wt-17βHSD10) increased both mitochondrial territory and fragmentation, while exacerbating ROS production. While we speculate that mitophagy and fusion-fission mechanisms underpin these effects, further studies should address these mechanisms in detail. While ROS activate mitophagy (Wang et al., 2012; Fan et al., 2019), this serves to reduce oxidative damage following IR injury (Livingston et al., 2019). Importantly, 17βHSD10 has been reported to induce mitochondrial elongation by abrogating Drp1 recruitment to mitochondria (Bertolin et al., 2015), while Drp1-dependent mitophagy ensures the clearance of damaged mitochondria (Zuo et al., 2014). Pharmacological or siRNA-mediated inhibition of the pathway causes decreased mitochondrial fragmentation, accumulation of damaged mitochondria through selective blocking of mitophagy and increased ROS generation following IR (Zuo et al., 2014), which could explain the currently observed phenotype. Although 17βHSD10 expression did not affect astrocytic survival immediately after the insult, the overexpressed 17βHSD10 activity is likely to potentiate mitochondrial damage in the long-term by simultaneously affecting ROS production, mitochondrial network morphology, mitophagy, and respiration.

Yan et al. (2000) showed that high levels of neuronal 17βHSD10 in a mouse model of ischemic stroke are associated with higher ATP, which correlated with increased acetyl-CoA flux, increased ketone body (β-hydroxybutyrate; BHB) utilization and better recovery of the animals. Although such adaptation facilitates short-term recovery, astrocytic up-regulation of fatty acid oxidation in low glucose conditions imposes higher load on mitochondria for metabolic adaptation and escalating ROS and associated damage (Polyzos et al., 2019). This further suggests that the enzymatic role of 17βHSD10 in β-oxidation of ketone bodies such as BHB (Yan et al., 2000) and short branch-chained fatty acids (Luo et al., 1995) is of key importance to astrocytic metabolism during glucose deprivation.

Cerebral ischemia also increases the risk of developing AD (Ballard et al., 2000; Pluta and Ułamek, 2008). In addition, there are multiple mechanisms proposed to drive AD pathology and among the key hallmarks are amyloid pathology (Karran et al., 2011; Hammond et al., 2020), complex brain metabolic dysfunction (Costantini et al., 2008) and mitochondrial aberrations (Swerdlow, 2018). Furthermore, Yan et al. (1999) found that both Aβ_(1-40)_ and Aβ_(1-42)_ bind to 17βHSD10 which inhibits the enzymatic activity of the protein. This interaction was identified as a central neurotoxic event in AD pathology and preventing this interaction was found to be neuroprotective in a mAPP mouse model of AD (Lustbader et al., 2004).

Consistent with early findings by He et al. (2005), we provided evidence that 17βHSD10 expression increases in cortical astrocytes exposed to an amyloid-rich environment. Furthermore, overexpressing the catalytically active (and not the mutant) version of the protein reduced astrocytic viability following amyloid treatment. This was accompanied by inhibition of overexpressed wt-17βHD10 activity, which was in line with findings by Yan et al. (1999) showing that Aβ_(1-42)_ inhibits the enzymatic activity of the protein and 17βHSD10 overexpression exacerbates amyloid pathology (Yan et al., 1999; Lustbader et al., 2004; Takuma et al., 2005). We also observed exacerbated ROS generation in amyloid-treated astrocytes overexpressing wt-17βHSD10. These effects are similar to observations in neurons from AD mice co-overexpressing mutant APP and 17βHSD10 (Tg mAPP/17βHSD10), which have increased ROS production, reduced ATP levels and compromised mitochondrial integrity (Takuma et al., 2005). Importantly, ROS production was inhibited by AG18051-mediated inhibition of 17βHSD10. Therefore, the novel generation of inhibitors which are currently being developed (Aitken et al., 2017, 2019; Boutin, et al., 2021; Fišar et al., 2021) hold promise for effectively modifying 17βHSD10-mediated effects in pathology.

In contrast to mitochondrial elongation following severe metabolic stress, mitochondrial fragmentation induced by Aβ_(1-42)_ was exacerbated by elevated wt-17βHSD10. It is important to note that while ischemia-reoxygenation potentiated the activity of the enzyme (which was associated with increased elongation), Aβ_(1-42)_ inhibited the activity of overexpressed wt-17βHSD10. Although mitochondrial fragmentation is a known consequence of amyloid-induced toxicity, there are multiple mechanisms that have been identified to drive this change, including elevated ROS, alterations in the levels of mitofusin 1 and 2 (Mfn1/2) levels, Opa1 (optic atrophy 1), and Tomm40, as well as dynamin-related protein 1 (Drp1) and CypD (Manczak et al., 2013; Owens et al., 2015; Park et al., 2015). It is yet to be confirmed which mechanism could be down-stream of the currently reported fragmentation. However, it is clear that the effects of amyloidogenic and metabolic stress on mitochondrial architecture and function in astrocytes are severely affected by alterations in 17βHSD10 enzymatic activity.

Overall, our data revealed that 17βHSD10 regulates mitochondrial function in astrocytes and alters their response to amyloidogenic and metabolic stress. We confirmed that the enzyme is expressed and active in astrocytes from different brain regions. Importantly, 17βHSD10 affects mitochondrial metabolism in astrocytes differently to what has been reported in cancer and neuronal models. Our results further showed that it was particularly the catalytic function of the protein that affects spare respiratory capacity in astrocytes, while 17βHSD10 chemical inhibition counters this effect. We further found that the protein bears multiple implications for astrocytic recovery, oxidative stress, and mitochondrial network morphology in stress conditions. In light of recent findings showing that astrocytic mitochondrial metabolism might be key to their metabolic flexibility and region-dependent vulnerability in neurodegeneration, our findings suggest that 17βHSD10 might be an important regulator. Another important question that remained beyond the scope of the current study is to find out whether 17βHSD10 might have different effects in astrocytes isolated from different brain regions, since the protein is upregulated only in cortical and hippocampal areas of AD brains.

Despite these pending questions, the current study highlights the role of astrocytic 17βHSD10 as a potential mediator of pathologic mechanisms and an exciting potential target for developing meaningful therapeutics.

10.1523/ENEURO.0040-22.2022.f1-4 Extended Data Figure 1-4 Mouse cerebellar astrocytes express 17βHSD10. High-magnification images of acute brain slices stained for 17βHSD10 (magenta) and GFAP (cyan) with colocalization shown in white. The first column presents the overlay of GFAP and 17βHSD10 staining. 17βHSD10 and GFAP staining alone are shown in the following two columns. The fourth column shows the outlines of the GFAP staining obtained in ImageJ through enhancing (0.25%) and equalizing the contrast of the image followed by the “find edges” function. The final column shows the overlays of the GFAP outlines and 17βHSD10 staining, facilitating the representation of the colocalization. Scale bar: 30 μm. Download Figure 1-4, EPS file.

10.1523/ENEURO.0040-22.2022.f1-3 Extended Data Figure 1-3 Mouse hippocampal astrocytes express 17βHSD10. High-magnification images of acute brain slices stained for 17βHSD10 (magenta) and GFAP (cyan) with colocalization shown in white. The first column presents the overlay of GFAP and 17βHSD10 staining. 17βHSD10 and GFAP staining alone are shown in the following two columns. The fourth column shows the outlines of the GFAP staining obtained in ImageJ through enhancing (0.25%) and equalizing the contrast of the image followed by the “find edges” function. The final column shows the overlays of the GFAP outlines and 17βHSD10 staining, facilitating the representation of the colocalization. Scale bar: 30 μm. Download Figure 1-3, EPS file.

## Synthesis

Reviewing Editor: Aniko Korosi, Universiteit van Amsterdam Decisions are customarily a result of the Reviewing Editor and the peer reviewers coming together and discussing their recommendations until a consensus is reached. When revisions are invited, a fact-based synthesis statement explaining their decision and outlining what is needed to prepare a revision will be listed below. The following reviewer(s) agreed to reveal their identity: Tamas Kozicz.

The authors have improved the manuscript, with a few comments left that should be addressed before publication.

- Figure 1-1: Could you please quantify the colocalisation?

- Please add the mixed model procedure to the statistics section in the methods

- Sentence 404: both wildtype and mutant?

- I think it would benefit the manuscript if the exact same abbreviations are consistently used throughout the manuscript for the different forms (endogenous, over expressed wild type and over expressed mutant) of 17bHSD10. For example, now sometimes it is wild type, sometimes wt-17bHSD10, or sometimes undefined (for example sentence 503).

- Sentence 566: missing figure reference

- Sentence 600: I find this somewhat overstated and would suggest the authors to rewrite
